# Point of Care Nucleic Acid Testing for SARS-CoV-2 in Hospitalized Patients: A Clinical Validation Trial and Implementation Study

**DOI:** 10.1016/j.xcrm.2020.100062

**Published:** 2020-07-15

**Authors:** Dami A. Collier, Sonny M. Assennato, Ben Warne, Nyarie Sithole, Katherine Sharrocks, Allyson Ritchie, Pooja Ravji, Matthew Routledge, Dominic Sparkes, Jordan Skittrall, Anna Smielewska, Isobel Ramsey, Neha Goel, Martin Curran, David Enoch, Rhys Tassell, Michelle Lineham, Devan Vaghela, Clare Leong, Hoi Ping Mok, John Bradley, Kenneth G.C. Smith, Vivienne Mendoza, Nikos Demiris, Martin Besser, Gordon Dougan, Paul J. Lehner, Mark J. Siedner, Hongyi Zhang, Claire S. Waddington, Helen Lee, Ravindra K. Gupta, The CITIID-NIHR COVID BioResource Collaboration, Stephen Baker, John Bradley, Gordon Dougan, Ian Goodfellow, Ravindra K. Gupta, Paul J. Lehner, Paul Lyons, Nicholas J. Matheson, Kenneth G.C. Smith, Mark Toshner, Michael P. Weekes, Nick Brown, Martin Curran, Surendra Palmar, Hongyi Zhang, David Enoch, Daniel Chapman, Ashley Shaw, Vivien Mendoza, Sherly Jose, Areti Bermperi, Julie Ann Zerrudo, Evgenia Kourampa, Caroline Saunders, Ranalie de Jesus, Jason Domingo, Ciro Pasquale, Bensi Vergese, Phoebe Vargas, Marivic Fabiculana, Marlyn Perales, Richard Skells, Lee Mynott, Elizabeth Blake, Amy Bates, Anne-laure Vallier, Alexandra Williams, David Phillips, Edmund Chiu, Alex Overhill, Nicola Ramenante, Jamal Sipple, Steven Frost, Helena Knock, Richard Hardy, Emily Foster, Fiona Davidson, Viona Rundell, Purity Bundi, Richmond Abeseabe, Sarah Clark, Isabel Vicente

**Affiliations:** 1 Division of Infection and Immunity, University College London, London WC1E 6BT, UK; 2 Cambridge Institute of Therapeutic Immunology & Infectious Disease (CITIID), Cambridge CB2 0AW, UK; 3 Diagnostics for the Real World EU, Chesterford Research Park, Saffron Walden CB10 1XL, UK; 4 Department of Infectious Diseases, Cambridge University NHS Hospitals Foundation Trust, Cambridge CB2 0QQ, UK; 5 Clinical Microbiology & Public Health Laboratory, Public Health England, Cambridge CB2 0QQ, UK; 6 POC Testing, Department of Pathology, Cambridge University Hospitals NHS Foundation Trust, Cambridge CB2 0QQ, UK; 7 Department of Medicine, University of Cambridge, Cambridge CB2 0AW, UK; 8 National Institutes for Health Research (NIHR) Cambridge Biomedical Research Centre, Cambridge CB2 0QQ, UK; 9 NIHR Cambridge Clinical Research Facility, Cambridge CB2 0QQ, UK; 10 Department of Statistics, Athens University of Economics and Business, 28is Oktovriou 76, 104 34 Athens, Greece; 11 Department of Haematology, Cambridge University Hospitals NHS Foundation Trust, Cambridge CB2 0QQ, UK; 12 Massachusetts General Hospital, Boston, MA, USA; 13 Harvard Medical School, Boston, MA, USA; 14 Africa Health Research Institute, Durban 4001, South Africa

**Keywords:** COVID-19, SARS-CoV-2, POC, point of care, diagnostic test, nosocomial, infection control

## Abstract

There is an urgent need for rapid SARS-CoV-2 testing in hospitals to limit nosocomial spread. We report an evaluation of point of care (POC) nucleic acid amplification testing (NAAT) in 149 participants with parallel combined nasal and throat swabbing for POC versus standard lab RT-PCR testing. Median time to result is 2.6 (IQR 2.3–4.8) versus 26.4 h (IQR 21.4–31.4, p < 0.001), with 32 (21.5%) positive and 117 (78.5%) negative. Cohen’s κ correlation between tests is 0.96 (95% CI 0.91–1.00). When comparing nearly 1,000 tests pre- and post-implementation, the median time to definitive bed placement from admission is 23.4 (8.6-41.9) versus 17.1 h (9.0–28.8), p = 0.02. Mean length of stay on COVID-19 “holding” wards is 58.5 versus 29.9 h (p < 0.001). POC testing increases isolation room availability, avoids bed closures, allows discharge to care homes, and expedites access to hospital procedures. POC testing could mitigate the impact of COVID-19 on hospital systems.

## Highlights

Point of care (POC) test highly sensitive and specific against RT-PCR
Time to result 2.6 h for POC versus 26.4 h for standard lab RT-PCR
SARS-CoV-2 POC test reduces median time-to-bed placement by ∼6 h
SARS-CoV-2 POC improves indices of hospital functioning and patient care

## Graphical Abstract

**Figure:**
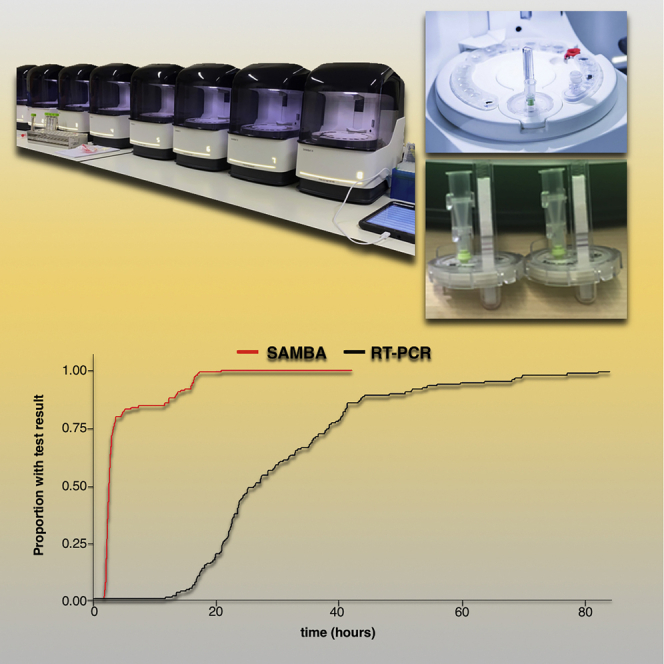

## Teaser

Collier et al. report testing and implementation of an accurate SARS-CoV-2 point of care nucleic acid amplification test that leads to faster time to definitive bed placement from the ER, release of isolation rooms, avoidance of bed closures, and expedited discharges to care homes and access to hospital investigations and procedures.

## Introduction

As of June 22, 2020, 9.0 million people have been infected with severe acute respiratory syndrome-coronavirus-2 (SARS-CoV-2), with >469,939 deaths and 40,000 deaths in the United Kingdom attributed to coronavirus disease 2019 (COVID-19).^1^ Current clinical testing for acute SARS-CoV-2 infection and infection risk relies on nucleic acid detection using reverse transcription polymerase chain reaction (RT-PCR) on nose and throat swabs.^2^^,^^3^ Antibodies to SARS-CoV-2 are detectable in only 50% by days 5–7^4^ and are therefore not suitable as a test for early infection, although they are useful in the second phase of illness, when virus detection wanes in upper respiratory tract samples.^3^^,^^5^ Antigen tests for COVID-19 diagnosis have performed poorly to date, and therefore nucleic acid detection remains the test of choice. Nucleic acid testing usually requires central laboratory testing with concomitant delays, and turnaround times are usually in excess of 24 h, and often days.^6^ Due to the diverse presentations of COVID-19,^7^ lack of a timely diagnosis can have serious consequences, including deadly nosocomial outbreaks.^8^

Screening hospital admissions rapidly is therefore critical to manage patient flow and limit the potential for nosocomial transmission.^9^^,^^10^ In the absence of a reliable point of care (POC) test, hospitals have resorted to creating bespoke care pathways to use isolation rooms most effectively for vulnerable patients.^11^ Finally, given care home outbreaks, there is also an urgent need to rapidly demonstrate COVID-19 status on discharge planning. This need for rapid and safe patient movement is likely to increase sharply in late 2020, when norovirus and influenza (with or without SARS-CoV-2) will likely compound the pressure on hospitals and isolation capacity, in particular. Such an approach would also relieve the pressure on hospital virology laboratories so that they can resume routine testing.

A number of near-patient tests have been described. Some have not performed well,^12^ and none have undergone testing under rigorous clinical trial conditions with real-world data on the impact on patient management.^13^, ^14^, ^15^, ^16^, ^17^ Thorough, prospective evaluation for a high-consequence pathogen such as SARS-CoV-2 is particularly important, given the risks related to false positives or negatives in the hospital setting.

SAMBA (simple amplification-based assay), an isothermal amplification-based platform, has been extensively field tested for HIV diagnostic applications in low resource settings,^18^^,^^19^ and has been adapted for use in SARS-CoV-2, with successful pre-clinical testing using synthetic standards and stored positive and negative clinical samples.^20^ Here, we present a prospective clinical validation trial comparing SAMBA II SARS-CoV-2 performance against the standard lab RT-PCR test in suspected COVID-19 cases presenting to hospitals, followed by an analysis of POC implementation in hospitals.

## Results

### Clinical Validation Study of SARS-CoV-2 POC Test

**Figure 1:**
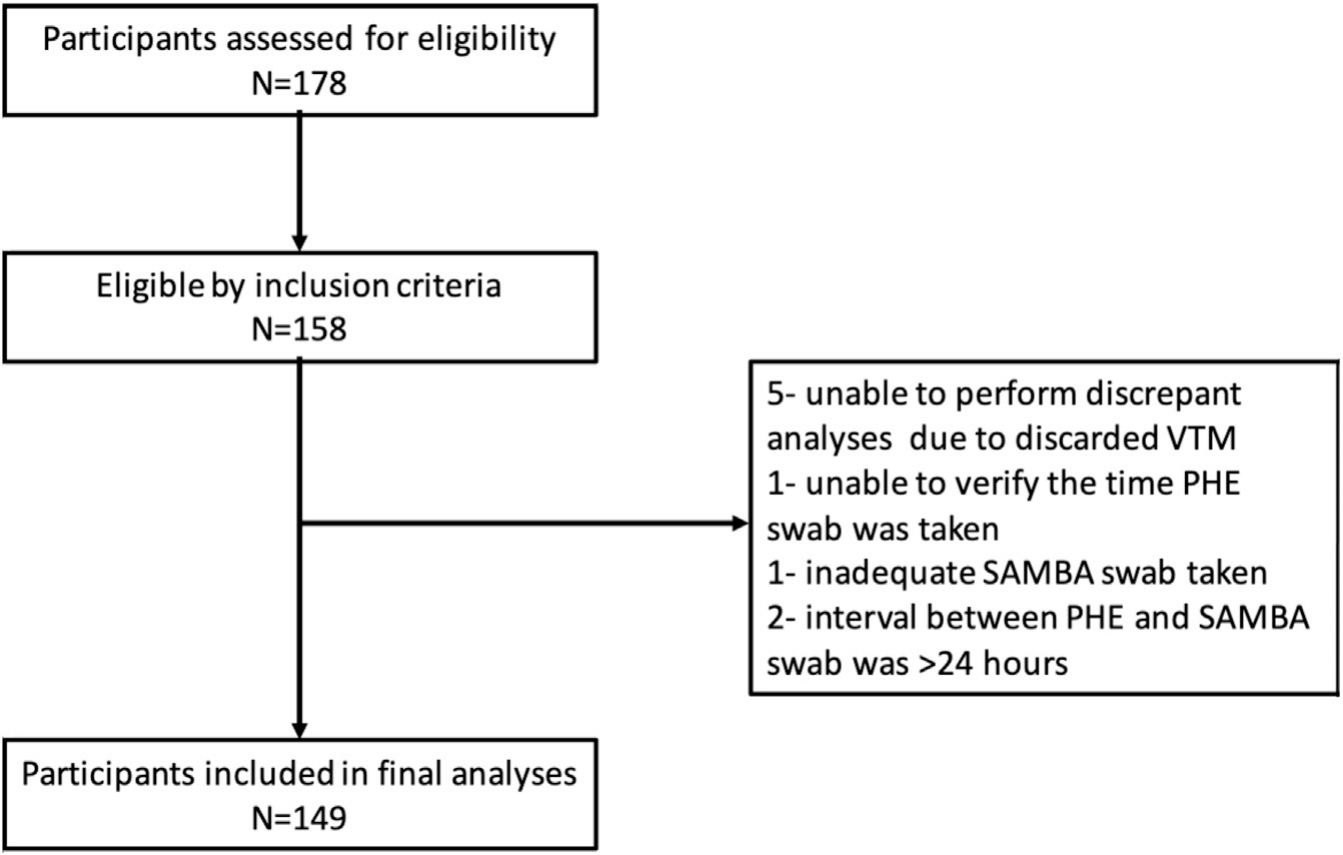
Prospective Clinical Study Flowchart Consolidated Standards of Reporting Trials (CONSORT) Diagram PHE, Public Health England; SAMBA, simple amplification-based assay; VTM, viral transport medium;.

**Figure 2:**
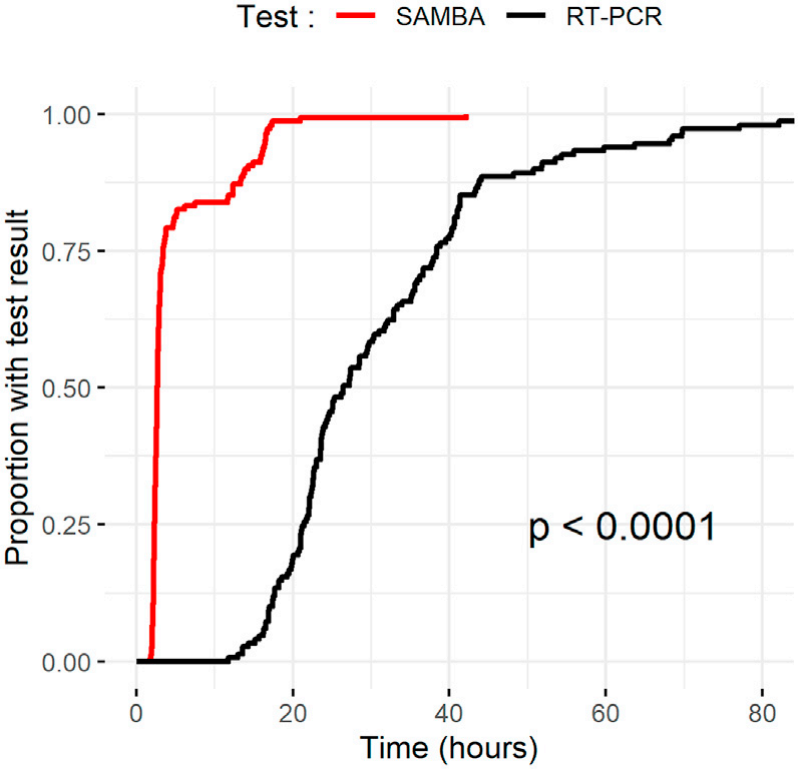
Kaplan-Meier Plot of the Time to Test Result under Clinical Validation Trial Conditions The time to test result in hours for the SAMBA II SARS-CoV-2 test (red) compared with the standard lab RT-PCR (black) (log rank test p < 0.001). RT-PCR, reverse transcriptase-polymerase chain reaction.

Of 178 screened patients, 149 met the eligibility criteria for inclusion in the clinical trial (Figure 1). The mean age was 62.7 years, and 47% were male. A total of 32/149 (21.6%) tested positive by the standard lab RT-PCR test. The mean temperature and respiratory rate were higher in the standard lab RT-PCR positive group (Table 1). The median duration of symptoms was 3 (interquartile range [IQR] 1.75–10.5) and 4 (IQR 2–13) days in standard lab RT-PCR positive and negative participants, respectively. There were 7 discrepant results between the POC and laboratory assays (7/149) after initial testing. The discrepancy analysis concluded that there was one false negative by the POC test, likely related to sampling variation, and no false positives (Table S1). The standard lab RT-PCR had one false negative in a participant with clinical and radiological evidence of disease. Cohen’s κ correlation between the 2 tests was 0.96, with a 95% confidence interval (CI) of 0.91–1.00. The effective sensitivity of the SAMBA II SARS-CoV-2 test as compared to the standard lab RT-PCR was 96.9% (95% CI 84.2–99.9), with a specificity of 100% (95% CI 96.9–100) (Table 2). POC testing (POCT) was associated with a shorter time from sampling to result (Figure 2); the median time to result was 2.6 h (IQR 2.3–4.8) for POCT and 26.4 h (IQR 21.4–31.4) for the standard lab RT-PCR test (p < 0.001).

**Table 1:** Baseline Characteristics of Prospective Participants in the COVIDx Trial

Variable	Negative	Positive	Total
**Age, y, n**	**117**	**32**	**149**

Mean (SD)	60.4 (19.8)	72.8 (17.8)	62.7 (20.0)
Median	62.5	75.5	63

**Gender (%)**			

Female	67/116 (58)	11/32 (34)	83/158 (53)
Male	49/116 (42)	21/32 (66)	75/158 (47)

**SpO**_**2**_**%**			

Mean (SD)	95.9 (3.20)	94.2 (4.23)	95.3 (3.78)
Median	97	95	96
Temperature, °C, mean (SD)	37.5 (0.914)	38.4 (1.030)	37.7 (1.015)
Respiratory rate/min, mean (SD)	20.2 (4.16)	23.4 (6.01)	21.1 (5.16)
Systolic blood pressure, mmHg, mean (SD)	136 (22.6)	137 (26.5)	137 (22.9)
Diastolic blood pressure, mmHg, mean (SD)	76.0 (12.7)	70.0 (10.2)	74.8 (12.3)
Lymphocyte count × 10^9^ cells/L, mean (SD)	1.42 (0.926)	1.08 (1.050)	1.26 (0.999)
Platelet count × 10^9^ cells/L, mean (SD)	270 (115.8)	216 (88.2)	244 (106.7)

COVID, coronavirus disease; SpO_2_, oxygen saturation.

**Table 2:** Accuracy of the SAMBA II SARS-CoV-2 Test Compared with Standard Lab RT-PCR Testing

	Standard RT-PCR Negative	Standard RT-PCR Positive	Total
SAMBA II SARS-CoV-2 Negative	116	1	117
SAMBA II SARS-CoV-2 Positive	1	31	32
Total	117	32	149

RT-PCR, reverse transcriptase-polymerase chain reaction; SAMBA, simple amplification-based assay; SARS-CoV-2, severe acute respiratory syndrome-coronavirus-2.

### SARS-CoV-2 POCT Implementation Study

A total of 992 SAMBA II SARS-CoV-2 tests were performed between May 2 and May 11, 2020 inclusive in 913 individuals. POCT was used for the following main indications: 59.8% of tests were used for newly hospitalized patients, and the remainder were used for pre-operative screening (11.3%), discharges to nursing homes (10.0%), in-hospital screening of new symptoms (9.7%), screening in asymptomatic patients requiring hospital admission screening (3.8%), and access to interventions such as dialysis and chemotherapy for high-risk patients (1.2%) (Table 3). The median time to result was 3.6 h (IQR 2.6–5.8). The rapid result from a POC test was deemed to have a beneficial clinical impact in 77.4% of patients who underwent the test (Table S2).

**Table 3:** Clinical and Demographic Data of 992 Tests in 913 Patients Who Had the SAMBA II SARS-CoV-2 Test in the Post-implementation Period

	(N) Individual Patients = 913/Tests = 992
Male gender (%)	n = 857/913
	389 (44.6)
Median age, y (IQR)	n = 909/913
	63 (37–79)
Duration of illness, days (IQR)	2 (1–7)

**SAMBA II SARS-CoV2 result (%)**	

Positive	42 (4.2)
Negative	950 (95.8)

**Triage at initial assessment (%)**	n = 966/992

Non-COVID-19 (green)	478 (49.5)
Possible COVID-19 (amber)	387 (40.0)
Likely COVID-19 (red)	101 (10.5)

**Inpatient transfer (%)**	n = 976/992

Yes	20 (2.0)
No	956 (98.0)

**Triage following SAMBA II SARS-CoV-2 result (%)**	n = 756/992

Non-COVID-19 (green)	600 (79.4)
Possible COVID-19 (amber)	88 (11.6)
Likely COVID-19 (red)	68 (9.0)

**Reason for SARS-CoV-2 test**	n = 970/992

Admission triage and placement	580 (59.8)
In-hospital triage and placement	94 (9.7)
Discharge to nursing home/carers	97 (10.0)
Pre-operative	110 (11.3)
Facilitate other investigations	12 (1.2)
Asymptomatic screening	37 (3.8)
Other	40 (4.1)

Note that some individuals had multiple admissions each with associated POC tests. COVID-19, coronavirus disease 2019; IQR, interquartile range; POC, point of care; SAMBA, simple amplification-based assay; SARS-CoV-2, severe acute respiratory syndrome-coronavirus-2.

**Figure 3:**
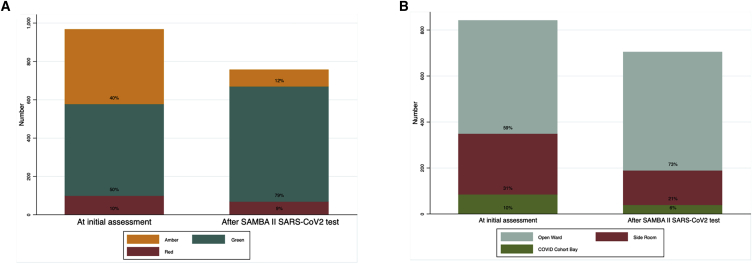
Impact of SAMBA II SARS-CoV-2 Testing on COVID Risk Stratification and Change in Use of Single-Occupancy Isolation Rooms (A) The assigned risk of COVID at initial assessment by a clinician at presentation and reassignment of COVID risk following the results of the SAMBA II SARS-CoV-2 test. Red, amber, and green represent high-, medium-, and low-risk clinical areas, respectively (p < 0.001 χ^2^ test). (B) The isolation type at initial assessment and following the results of the SAMBA II SARS-CoV-2 test (p < 0.001 χ^2^ test).

POCT with negative results allowed a significant increase in the number of patients able to move to “green” non-COVID-19 areas (green status [478/966] 49.5% before the test and [600/756] 79.4% afterward, p < 0.001). The numbers in “amber” areas (possible COVID-19) fell reciprocally (Figure 3A) (40% on amber before test and 11.6% after test, p < 0.001), thereby allowing quicker access to potentially lifesaving procedures such as computed tomography (CT) angiography or cardiac monitoring (Table S5). We observed a concomitant decrease in the use of single-occupancy rooms among those tested for new in-hospital COVID-19 symptoms, from 30.8% before to 21.2% (p = 0.03) after the POC test result (Figure 3B). Eleven bay closures were prevented with POCT overall, with each bay having an average of 6 beds.

We then examined the clinical utility of POCT for a range of indications (Table S2).

#### Emergency Admissions

Rapid SAMBA II SARS-CoV-2 POCT was deemed beneficial in 436 (75.8%) tests performed at presentation to the emergency department (ED) or the acute admission ward. In 12 instances, a negative POC result did not change the initial risk assessment, isolation, or clinical management due to a high clinical suspicion of COVID-19. It is well known that the diagnosis of COVID-19 is complicated in a number of patients who have negative PCR nose and throat swabs, frequently after the first week of illness, when SARS-CoV-2 antibody responses become detectable.^4^ In the remaining 121 (21.2%) tests in which no clinical benefit was derived, the reasons for this were patients being discharged home from the ED before the result became available, patients being triaged and moved to a ward before the results were available, and patients having a previous recent SARS-CoV-2 test result.

#### Pre-operative Testing

A total of 110 (11.3%) tests were performed in advance of surgical procedures, partly for infection control purposes, but mainly to screen patients in light of data demonstrating increased peri-operative mortality associated with COVID-19.^21^ POC tests were deemed to have resulted in clinical benefit attributable to the rapid result (Table 3) in 106/110 (96.3%) instances. SAMBA II SARS-CoV-2 testing facilitated surgical interventions, including exploratory laparotomy, eye and maxillofacial surgery, solid organ transplants, and caesarean sections.

#### Discharge to Care Home or with a Care Package

Nursing homes came to be recognized as hotspots for COVID-19 transmission, and at the end of April 2020, national policy mandated a SARS-CoV-2 swab <48 h before discharge to a nursing home or a setting where an individual was visited by caregivers. SAMBA II SARS-CoV-2 testing was successfully used to facilitate discharge in 76/96 (79.2%) instances. In the remaining 20.8%, alternative reasons were identified in the discharge pathway, which resulted in delays that required another test to meet the hospital’s discharge policy.

#### Prevention of Healthcare-Associated Infection

A SAMBA II POC test was carried out in 94 patients for the purpose of in-hospital triage and placement; 81 of these had sufficient data to determine the impact of the SAMBA II SARS-CoV-2 test. The test was beneficial in 55.6% (45/81), allowing the patient to remain in a low-risk open ward in 68.9% (31/45) of instances, movement out of a side room in 17.8% (8/45), and avoiding bay closures in 13.3% (6/45). In the remaining 44.4% (36/81) of instances in which no beneficial impact was found, 7 of these had a previous recent test result, 2 of which were known to be positive, and a SAMBA positive result had no further impact. In 4 instances, the patient had been moved before the result returning as clinical suspicion of COVID-19 was high, leading to triage before the result being known; in 8 instances, there was no documented indication; and in the rest, SAMBA II SARS-CoV-2 testing did not alter management.

**Figure 4:**
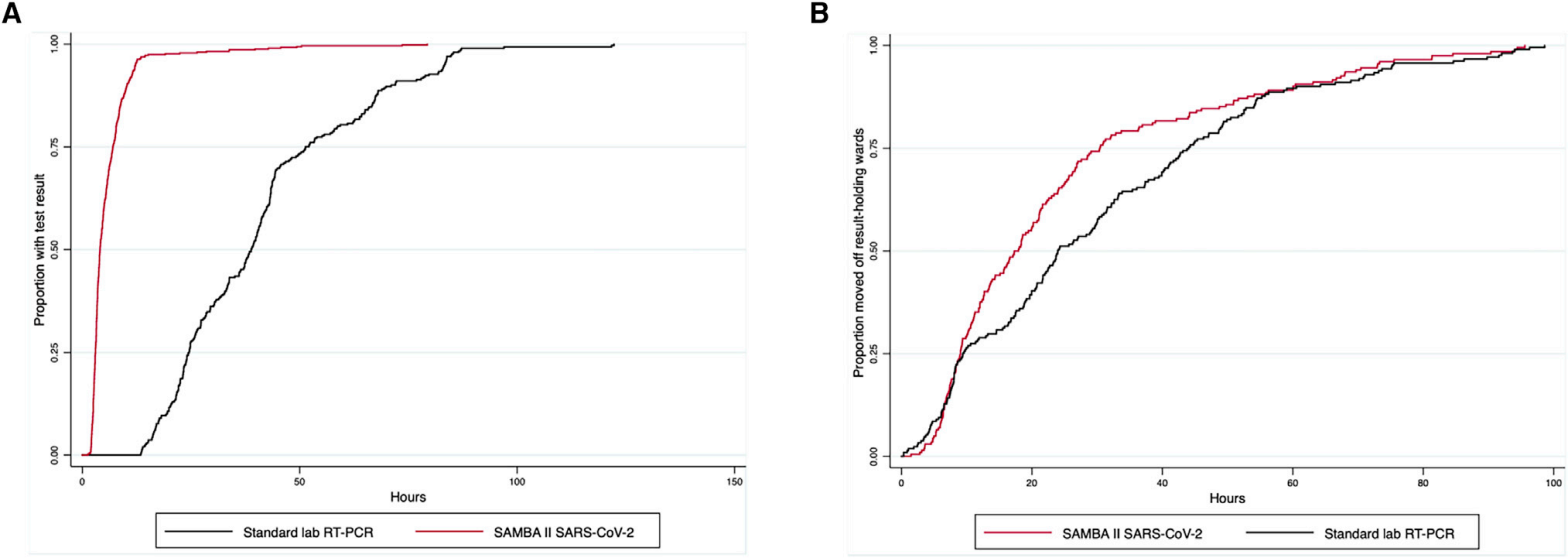
Kaplan-Meier Plots of the Time to Test Results and Definitive Ward Move Comparing SAMBA II SARS-CoV-2 Test in the Post-implementation Period with the Standard Lab RT-PCR in the Pre-implementation Period (A) The time to test result in hours for the SAMBA II SARS-CoV-2 test (red) compared with the standard lab RT-PCR (black) (log rank test p < 0.001). (B) The time to definitive ward move for SAMBA II SARS-CoV-2 POC test (red) compared with the standard lab RT-PCR (black) (log rank test p = 0.02).

Next, we compared clinical outcomes in the 10 days before and following SAMBA II SARS-CoV-2 introduction. Duplicate tests in the same admission episode were excluded. We identified 561 tests in 388 individuals tested using the standard laboratory RT-PCR in the 10 days before SAMBA II SARS-CoV-2 introduction, and compared them with 913 tests done in 799 individuals using the POC test in the 10 days post- SAMBA II SARS-CoV-2 introduction. Demographic characteristics of both groups were similar. Clinical factors were different, which reflects the timeline of the pandemic; the proportion of positive tests, mortality, and presumed risk of COVID-19 was lower in the post-implementation period (Table S3). The time from sample to test result fell dramatically (35.9 h (23.8–48.9) to 3.8 h (2.7–6.0), p < 0.0001; Figure 4A shows Kaplan-Meier analysis). The time to definitive ward move from admission also decreased significantly after SAMBA II SARS-CoV-2 introduction (23.4 h (8.6–41.9) to 17.1 h (9.0–28.8), p = 0.02; Figure 4B shows Kaplan-Meier analysis). The Cox proportional hazards regression model showed that even after mutually adjusting for age, gender, quick sequential organ failure assessment (qSOFA) score, National Early Warning Score 2 (NEWS2), and Charlson Comorbidity Index (CCI), the SAMBA II SARS-CoV-2 test was independently associated with the shorter time to definitive bed placement from admission (hazard ratio [HR] 1.25 [95% CI 1.02–1.53], p = 0.03). Other significant associations were younger age and NEWS2 medium risk score (Table S4). Finally, mean length of stay on a COVID-19 result wait-holding ward decreased from 58.5 to 29.9 h (p < 0.001) compared to the 10 days pre-implementation.

## Discussion

Here, we report the impact of rapid POC molecular SARS-CoV-2 testing for the diagnosis of COVID-19 infection in a high-need hospital setting. These data demonstrate that POCT can be reliable and accurate and provide clinicians with much quicker results compared to the current standard of care test. Furthermore, we demonstrate that routine use of this test had a real-world impact on patient care and safety.

The POC SAMBA II SARS-CoV-2 nucleic acid test was compared to a reference RT-PCR test—the standard of care—using combined nasal and throat swabs from participants presenting to hospitals with a possible diagnosis of COVID-19. Study participants were representative of United Kingdom COVID-19 patients,^22^ and we found that the concordance between the tests was extremely high, with a Cohen κ coefficient of 0.96. When the standard lab RT-PCR test was referenced as a gold standard, the sensitivity of SAMBA was 96.9% and the specificity was 100%. The median time from swab to result was 2.6 h for SAMBA II as compared with 26.4 h for RT-PCR (p < 0.001). Although the Hologic Panther Fusion platform used for the standard lab RT-PCR test has a turnaround time of ∼3 h post-RNA extraction, the median turnaround times of >24 h in our study reflects the logistical challenges of performing these tests at the peak of the epidemic in our hospital. Specimens were handled in biosafety level 3 (BSL 3) laboratory and batch runs, which created a significant delay. This aspect of delay was overcome by the SAMBA II platform, which uses an inactivation buffer, thereby avoiding the requirement for viral transport media and BSL 2 and 3 facilities.

Patient placement during the COVID-19 pandemic has been a significant challenge and has had a great impact on our ability to maintain patient flow and safety in the hospital. The trial data on SAMBA II raised the prospect of addressing these problems. Our hospital switched from standard lab RT-PCR testing to SAMBA II for in-hospital testing immediately following the end of the validation study, providing an opportunity to prospectively evaluate almost 1,000 tests performed over 10 consecutive days. Most of the tests were performed on new admissions to the hospital and replicated the significant reduction in test turnaround time observed in the clinical validation trial.

POC was also used to investigate newly symptomatic patients in hospital to rationalize our limited isolation rooms, and also to rapidly identify new COVID-19 cases, with appropriate infection control and prevention of nosocomial outbreaks.^10^ Inappropriate isolation is a large drain on staff and resources due to the need for repeated deep cleaning, additional personal protective equipment (PPE) utilization and the distress and risk to patients from repeated bed moves.^23^ As expected, we observed a significant increase in the availability of isolation or single-occupancy rooms following POC introduction, and patients who tested negative were able to be placed in low-risk areas of the hospital and have interventions and procedures expedited.

We found that 11 ward closures were prevented in the 10-day post-implementation phase by there being negative tests in symptomatic hospital patients. Closed surgical bays in particular can result in the cancellation of operations, as well as significant financial losses to hospitals. Following this analysis, hospital guidelines will be adapted to recommend waiting for SAMBA test results before moving patients into isolation or closing bays.

When we performed a formal implementation impact analysis using 10-day windows on either side of May 2, 2020, we found that time to definitive ward move from admission decreased significantly after the introduction of SAMBA II SARS-CoV-2 testing, and length of stay on the main holding ward where test results were awaited also fell significantly, which is consistent with more rapid and accurate patient movement.

Although we did not conduct a cost-benefit analysis in this study, the utilization of POCT in acute settings for other respiratory viruses has been shown to be cost-effective.^24^ Given the morbidity and mortality associated with COVID-19 and the disruption in healthcare provision caused by this pandemic, we anticipate that SAMBA II SARS-CoV-2 implementation is also likely to be a cost-effective intervention through reductions in delayed discharge, nosocomial transmission, and unnecessary use of PPE. Formal economic analyses of POCT implementation in pandemic settings are required.

SAMBA II SARS-CoV-2 testing is being implemented in a very limited number of hospitals, but there is an urgent need for POC capacity in care homes, prisons, and other establishments. A POC platform also has the potential to reduce disparities between secondary and tertiary medical centers that have specialized virology laboratories, and ensures equitable access to timely SARS-CoV-2 testing results. SAMBA II machines are already in use in Uganda, Zimbabwe, and Kenya for HIV testing and monitoring. If scale-up can be achieved in those settings, POCT could be vital for controlling COVID-19 in sub-Saharan Africa,^8^ and our data will inform its optimal use.^25^

Finally, based on the data presented, we predict that the implementation of POCT for SARS-CoV-2 could have a critical impact on the hospital management of suspected COVID-19 cases, particularly in the context of influenza and norovirus seasons.

### Limitations of Study

The clinical test validation component was limited by the fact that the same swab could not be tested on the two platforms being compared. This raised an issue of two separate samples being tested on the two assays. Nonetheless, we identified only two cases in which the sampling may have explained discrepant results. In addition, the SAMBA II SARS-CoV-2 test is not capable of providing viral load or cycle threshold values for more nuanced analysis. The implementation phase took place 6 weeks into the United Kingdom lockdown, at a time when the rate of new infections had reduced substantially across the country. Nonetheless, the study highlights the importance of rapid test results in the COVID-19 era, regardless of the outcome of the test results. It should also be borne in mind that nucleic acid tests on nose and throat swabs can be negative in COVID-19 disease, particularly when presentation to the hospital occurs beyond 7 days.^26^ However, for general hospital infection control purposes, nose and throat nucleic acid detection is seen to be appropriate for infection control and triaging purposes. Finally, the use of a proprietary inactivation buffer may limit the generalizability of the platform, particularly since supply shortages are a major problem in COVID-19 diagnostic assays.

## STAR★Methods

### Key Resources Table

**Table:** 

REAGENT or RESOURCE	SOURCE	IDENTIFIER
**Biological Samples**		

Participants combined nose and throat swab	This study	N/A

**Critical Commercial Assays**		

SAMBA II SARS-CoV-2 test	Diagnostics for the real World	Cat# 8500-12
SARS-CoV-2 RT-PCR in-house test on was performed on QIAGEN Roto gene platform	QIAGEN	

**Software and Algorithms**		

STATA version 13	STATA	https://www.stata.com/order/download-details/
R 2.6.3	The R project	https://www.r-project.org/

### Resource Availability

#### Lead Contact

Further information should be directed to and will be fulfilled by the Lead Contact, Ravindra Gupta rkg20@cam.ac.uk.

#### Materials Availability

This study did not generate new unique reagents.

#### Data and Code Availability

Raw anonymised data are available from the lead contact.

### Experimental Model and Subject Details

The study was conducted in two phases; a clinical validation phase followed by an implementation phase.

#### Clinical validation study

The COVIDx Study was a prospective, comparative, real world trial of SAMBA II SARS-CoV-2 point of care testing compared to the standard lab RT-PCR test in participants admitted to Cambridge University Hospitals NHS Foundation Trust (CUH) with a possible diagnosis of COVID-19 (Data S1). CUH is a 1200-bed hospital providing secondary care to a population of 580,000 people in Cambridge and the surrounding area, as well as tertiary referral services to the East of England.

Recruitment started two weeks into the national lockdown implemented by the UK government in response to the pandemic. Eligible consecutive participants were recruited during 12-hour day shifts over a duration of 4 weeks from the 6^th^ of April 2020 to the 2^nd^ of May 2020. The prevalence of PCR positive SARS-CoV-2 infection among in-hospital patients in CUH decreased over the course of the study from 14.8% to 3.1% from week 1 to week 4 of the study. This reflected the background prevalence in Cambridgeshire which decreased from 17.9 per 100 000 population to 14.6 per 100 000 population in weeks 1 to 4 of the study^27^. We recruited adults (> 16 years old) presenting to the emergency department or acute medical assessment unit as a possible case of COVID-19 infection. This included participants who met the Public Heath England (PHE) definition of a possible COVID-19 case: any individual requiring hospital admission and has any of: clinical or radiological evidence of pneumonia, or acute respiratory distress syndrome, or an influenza like illness (history of fever and at least one of the following respiratory symptoms, which must be of acute onset- persistent cough (with or without sputum), hoarseness, nasal discharge or congestion, shortness of breath, sore throat, wheezing, sneezing. This definition was later expanded to include any adult requiring hospital admission and who was symptomatic of SARS-nCOV2 infection, demonstrated by clinical or radiological findings. This was done due to the changing landscape of the COVID-19 epidemic and emergence of new symptoms such as anosmia and diarrhea. This protocol amendment was applied after 77% of participants had been enrolled. The inclusion criteria were later expanded to include any adult requiring hospital admission and who was symptomatic of SARS-CoV-2 infection, demonstrated by clinical or radiological findings. This was done due to the changing landscape of the COVID-19 epidemic and emergence of new symptoms such as anosmia and diarrhea. Exclusion criteria included not having the standard lab RT-PCR test applied within an 18-hour window of SAMBA II SARS-CoV-2 test and those unwilling or unable to comply with study swabbing procedures.

#### Clinical Implementation Study

Following the completion of the COVIDx validation study (May 1^st^ 2020) and demonstration of performance equivalent to the reference standard test, the hospital switched from standard lab RT-PCR testing to use of SAMBA II for in-hospital testing due to its shorter turnaround time. There were no changes in the testing criteria over the implementation study. Twenty SAMBA II machines were operationalised by the CUH POC testing team, each machine capable of performing around 10-15 tests per day. To evaluate the real-world impact of SAMBA on clinical care, we retrospectively gathered data on clinically relevant endpoints from electronic patient records over a ten-day period before and after introduction of the SAMBA test for all patients who underwent COVID-19 testing.

All patients who underwent COVID-19 testing in a 10 day period before and after introduction of the SAMBA II SARS-CoV-2 test were included. Participants were identified from testing reports from the EPIC electronic hospital records system. Clinical and hospital activity data were obtained from the same source. The determination of whether the SAMBA II SARS-CoV-2 test was of benefit or not was made by a clinician who reviewed each participants’ clinical notes. The test was deemed to be of benefit if the result facilitated a clinical decision which would otherwise have been delayed had a rapid test not been available.

### Method Details

#### Test methods

Participants in the COVIDx trial were tested using SAMBA II SARS-CoV-2 on a combined nasal/throat swab within 18 hours of a similar swab for the standard lab RT-PCR test. The index test is the SAMBA II SARS-CoV-2 test - a nucleic acid amplification test (NAAT) which uses nucleic acid sequence based amplification to detect SARS-CoV-2 RNA from throat and nose swab specimens collected by dry sterile swab and inactivated in a proprietary inactivation buffer prior to analyses. This obviates the need for a BSL3 laboratory for specimen handling or viral extraction. The SAMBA II SARS-CoV-2 targets 2 genes- Orf1 and the E genes. The limit of detection (LoD) of the SAMBA II SARS-CoV-2 Test is 250 copies/ml^20^. The SAMBA II instrument system consists of the SAMBA II Assay Module (P/N I19-0006-AM) and the SAMBA II Tablet Module (P/N I19-0006-TM). The assay module sits on a bench top at room temperature and has an approximate size of 20cmx20cmx20cm. The SAMBA II SARS-CoV- 2 test contains all materials required for extraction of viral nucleic acid from the specimen, amplification of the nucleic acid target and the detection of the amplification products. All cartridges required to test one sample using SAMBA II SARS-CoV-2 test are packaged in a One Test Set bag. The assay module is able to process one sample at a time and takes 90 minutes to run. One assay module is able to perform 10-15 tests in a 24-hour period.

There is currently no gold standard for the diagnosis of COVID-19. In lieu of a gold standard the reference standard used for this study is an in-house RT- PCR test developed in the public health England (PHE) laboratory at CUH with a LOD of 320 copies/ml. This test was performed on the QIAGEN Roto gene platform which gives a result in 3 hours and able to perform 100 samples at a time. Specimens were handled in at BSL 3 laboratory and batch run, both of which contributed to increased TAT (test turnaround time).

Indeterminate SAMBA II SARS-CoV-2 tests occurred if the positive control line was absent on the test strip and were repeated with a 1:2 dilution of sample to inactivation buffer according to manufacturer standard operating procedures until a valid result was obtained.

For lab RT-PCR, a dilution of the MS2 bacteriophage was added to all samples prior to the extraction step to act as an internal/inhibition control. In the result of internal control failure, the result was classed as “invalid.” The results of the SAMBA II SARS-CoV-2 was not known to the assessors of the standard lab RT-PCR.

#### Data Collection

Demographic and clinical data were obtained at presentation from the hospital’s electronic patient records (EPIC) and entered into anonymised case report forms on the MACRO electronic database. Biological specimens from a combined nose and throat swab were collected and stored by research nurses. Results were not made available to clinical teams during the study. The primary outcome measures were time to result, concordance with the standard lab RT-PCR test and sensitivity/specificity of the SAMBA II SARS-CoV-2 test.

#### Ethical approval

The protocol was approved by the East of England - Essex Research Ethics Committee. HRA and Health and Care Research Wales (HCRW) approval was received. Verbal informed consent was obtained from all participants or in the case of participants without capacity, from a consultant nominee who was involved in their clinical care but independent from the research team. The implementation study was registered as a service evaluation with Cambridge University Hospitals NHS Foundation Trust.

Patients or the public were not involved in the design, or conduct, or reporting, or dissemination plans of our research. There are no plans to directly feedback the results to participants.

### Quantification and Statistical Analysis

#### Clinical Validation Study

We assumed a target sensitivity of 0.95 and disease prevalence of 15%. Using a 5% significance level and allowing for an error of 10% gave a required sample size of 122. Participants with missing SAMBA II SARS-CoV-2 or standard lab RT-PCR tests result were excluded from the analyses. Descriptive analyses of clinical and demographic data are presented as median and interquartile range (IQR) when continuous and as frequency and proportion (%) when categorical. The difference in continuous and categorical data were tested using Wilcoxon rank sum and Chi-square test respectively. Agreement between the two tests was assessed using Cohen’s kappa, a correlation-like measure which accounts for agreement by chance alone, in which case κ = 0, while κ = 1 and κ = −1 correspond to perfect agreement and completely discordant pairs respectively. Sensitivity and specificity of SAMBA II SARS-CoV-2 test were presented with exact Clopper-Pearson 95% confidence intervals due to estimates being near 1. Kaplan Meier survival analysis was used to compare time to result for the two tests, with log rank testing. Analysis was done using R and STATA version 13.

#### Clinical Implementation Study

The main study outcomes in the implementation study were the indication for SAMBA II SARS-CoV-2 test and perceived impact. Secondary outcomes were time to definitive patient triage from the emergency department (ED), time spent on COVID-19 holding wards, bay closures avoided, proportions of patients in isolation rooms following test and proportions of patients able to be moved to COVID-19 negative open wards following test.

Descriptive analyses of clinical and demographic data are presented as median (IQR) when continuous and frequency (%) when categorical. Difference in continuous variables between the pre and post implementation groups were assessed using the Wilcoxon rank sum tests and difference in categories and proportion were assessed using the Chi-square test or test of proportions. Kaplan Meier survival analysis was used to compare time to result and time to definitive bed placement from admission for the two tests, with log rank testing. Cox proportional hazards regression was used to estimate the hazard ratio (HR) of the associations between time to definitive bed placement and participant clinical and demographic factors. In the final multivariable model, mutually adjusted estimates of the HRs were determined by including those factors with evidence of an association in the univariable analysis and a p value of < 0.1. Although gender was not significantly associated with time to definitive bed placement in the univariable analysis, it was kept in the final model as it was an *a priori* specified confounder. Analysis was done using STATA version 13.

### Additional Resources

COVIDx was registered with the ClinicalTrials.gov Identifier NCT04326387.

## Supplemental Information

Document S1. Tables S1–S5 and Data S1

Document S2. Article plus Supplemental Information
