# Supplementary material for: Point of Care Nucleic Acid Testing for SARS-CoV-2 in Hospitalized Patients: A Clinical Validation Trial and Implementation Study
Source: Cell Rep Med. 2020 Jul 15;1(5):100062. doi: 10.1016/j.xcrm.2020.100062 (PMC7362826; doi:10.1016/j.xcrm.2020.100062)
Supplement: Document S1. Tables S1–S5 and Data S1 [file mmc1.pdf]

Cell Reports Medicine, Volume *1*

Supplemental Information

Point of Care Nucleic Acid Testing for SARS-CoV-2 in Hospitalized Patients: A Clinical Validation Trial and Implementation Study

Dami A. Collier, Sonny M. Assennato, Ben Warne, Nyarie Sithole, Katherine Sharrocks, Allyson Ritchie, Pooja Ravji, Matthew Routledge, Dominic Sparkes, Jordan Skittrall, Anna Smielewska, Isobel Ramsey, Neha Goel, Martin Curran, David Enoch, Rhys Tassell, Michelle Lineham, Devan Vaghela, Clare Leong, Hoi Ping Mok, John Bradley, Kenneth G.C. Smith, Vivienne Mendoza, Nikos Demiris, Martin Besser, Gordon Dougan, Paul J. Lehner, Mark J. Siedner, Hongyi Zhang, Claire S. Waddington, Helen Lee, Ravindra K. Gupta, and The CITIID-NIHR COVID BioResource Collaboration

	POC SAMBA swab			Lab RT-PCR swab				
ID	SAMBA (initial result)	SAMBA (repeat result)	Lab RT-PCR result	Lab (initial result)	Lab RdRp/E (Ct cycle)	SAMBA data	Clinical COVID-19 impression	Final result
1	Neg	Neg		36	Neg/Neg		Neg	Neg
2	Neg	Neg	Neg/Neg	34	Neg/33	Neg	Pos	Pos
3	Pos	Pos		Neg	Neg/32		Pos	Pos
4	Neg	Neg		36	Neg/Neg		Neg	Neg
5	Pos	Pos	31/23	Neg	Neg	Neg	Pos	Pos
6	Pos	Pos		Neg	33/34		Pos	Pos
7	Neg	Neg		31	Neg/Neg		Neg	Neg

Supplementary Table 1, related to table 2: Discrepancy analysis of seven samples. Instances where one SARS-Co-V2 nucleic acid test showed a different result from the other, both stored samples were identified and re-tested with the original test as well as the alternative. For the lab method a Ct cycle >36 is considered negative

	(N) Individual patients=913/ Tests=992
**Impact of test (%)**	N=970/992
**Bed placement at admission**	271(28.0)
**Facilitate discharge to another inpatient facility**	10 (1.0)
**Release of a side room**	32 (3.3)
**Expedited discharge**	100 (10.3)
**Expedited discharge to a nursing home/carers**	58 (6.0)
**Expedited surgery and other interventions**	128 (13.2)
**Safe to remain or move to a green ward**	112 (11.6)
**Avoided a bay closure**	11 (1.1)
**Facilitated a planned admission**	7 (0.7)
**No perceived impact**	228 (23.5)
**Other**	13 (1.3)

Supplementary Table 2, related to table 3: Perceived impact of SAMBA II SARS-CoV-2 testing.

	Pre-implementation<br>Standard PHE RT-PCR test<br>N= 561 in 388 persons	Post-implementation<br>SAMBA II SARS-CoV-2 test<br>N=913 in 799 persons	P value
**Gender (%)**<br>Male<br>Female	 197 (50.8)<br>191 (49.2)	 364(45.6)<br>434 (54.4)	 0.10[a]
**Median age** ***years (IQR)***	63.0 (42.0-79.5)	61.0 (36.0-78.0)	0.02[b]
**Acute Admission (%)**<br>Yes<br>No	 403 (71.8)<br>158 (28.2)	 615 (67.4)<br>298 (32.6)	 0.07[a]
**SARS-CoV2 result (%)**<br>Positive<br>Negative	 49 (8.7)<br>512 (91.3)	 39 (4.3)<br>874 (95.7)	 <0.001[a]
**Died (%)**<br>Yes	 28 (7.2)	 27 (3.4)	 0.003[a]
**Median length of admission** ***days (IQR)***	4.4 (1.1-10.8)	2.9 (0.9-7.3)	<0.0001[b]
**Triage at initial assessment (%)**<br>non-COVID-19 (green)<br>possible COVID-19 (amber)<br>likely COVID-19 (red)	N=544/561<br>249 (45.8)<br>244 (44.9)<br>51 (9.4)	N=856/913<br>450 (52.6)<br>349 (40.8)<br>57 (6.7)	 0.02[a]
**Median time to test result** ***hours (IQR)***	N=544/561<br>35.9 (23.8-48.6)	N=655/913<br>3.8 (2.7-6.0)	 <0.0001[b]
**Median time to definitive bed placement from admission** ***hours (IQR)***	N=160/561<br>23.4 (8.6 to 41.9)	N=267/913<br>17.1 (9.0-28.8)	 0.02[b]
**qSOFA score (%)**<br>0-1<br>2-3	N=551/561<br>513 (93.1)<br>38 (6.9)	N=903/913<br>851 (94.2)<br>52 (5.8)	 0.38[a]
**NEWS2 score (%)**<br>0-4 Low risk<br>5-6 Medium risk<br>>7 High Risk	N=555/561<br>407 (73.3)<br>82 (12.9)<br>66 (11.9)	N=906/913<br>711 (78.5)<br>107 (11.8)<br>88 (9.7)	 0.08[a]
**CCI score (%)**<br>< 4<br>>/=4	N=560/561<br>470 (83.9)<br>90 (16.1)	N=912/913<br>782 (85.8)<br>130 (14.2)	 0.34[a]

Supplementary table 3, related to figure 4: Clinical and demographic data of patients who had the standard PHE RT-PCR test in the pre-implementation period from 22nd of April 2020 till the 1st of May 2020 and those who had the SAMBA II CoV2 test in the post-implementation period from the 2nd of May 2020 till the 11th of May 2020. Duplicate tests during the same admission period were excluded. qSOFA- Quick sequential organ failure assessment score, NEWS2- National early warning score 2, CCI- Charlson Comorbidity Index

[a] Chi-square test
[b] Wilcoxon rank sum test

	Univariable model‡				Multivariable model‡	
	Events/ Follow up time§	Rateδ	HR (95% CI)	P value	HR (95% CI)	P value
ARS-CoV-2 Test						
Standard lab RT-PCR	211/64	3.31 (2.88-3.78)	1	0.01*	1	0.03*
SAMBA SARS-Cov-2	201/49	4.04 (3.54-4.67)	1.27 (1.05-1.55)		1.25 (1.02-1.53)	
ender						
Female	231/63	3.64 (3.20-4.15)	1	0.85	1	0.94
Male	181/50	3.63 (3.14-4.20)	0.98 (0.81-1.20)		1.01 (0.82-1.23)	
ge group (years)						
81-119	105/40	2.66 (2.19-3.21)	1		1	
65-80	96/31	3.11 (2.55-3.81)	1.17 (0.89-1.55)	0.26	1.29 (0.97-1.71)	0.08
42-64	125/28	4.54 (3.81-5.41)	1.84 (1.42-2.39)	<0.001*	1.83 (1.40-2.40)	<0.001*
0-41	87/16	5.53 (4.48-6.82)	2.43 (1.82-3.25)	<0.001*	2.51 (1.86-2.29)	<0.001*
SoFA score						
2-3	18/8.2	2.20 (1.39-3.50)	1	0.01*	1	0.12
0-1	388/100	3.74 (3.39-4.13)	1.83 (1.14-2.94)		1.54 (0.89-2.66)	
EWS2 score						
>7 High Risk	36/12	2.89 (2.08-4.00)	1		1	
5-6 Medium risk	54/20	2.64 (2.02-3.44)	0.85 (0.55-1.29)	0.44	0.58 (0.36- 0.92)	0.02*
0-4 Low risk	318/80	3.98 (3.57-4.44)	1.42 (1.01-2.01)	0.05*	0.92 (0.61-1.39)	0.69
CI score						
≦ 3	354/94	3.76 (3.38-4.17)	1			
≧ 4	57/19	3.07 (2.36-3.97)	0.79 (0.60-1.05)	0.12		

Supplementary Table 4, related to Figure 4: Multivariable analyses using Cox proportional hazards regression of the effect of SARS-CoV-2 test type on time to definitive bed placement for patients presenting for emergency care in accident and emergency and acute admissions departments. The standard PHE RT-PCR test was used in the pre-implementation period from 22nd of April 2020 till the 1st of May 2020 and the SAMBA II CoV2 test in the post-implementation period from the 2nd of May 2020 till the 11th of May 2020. Only the first test done by each participant in both phases of was included. Only patients who were admitted were included. qSOFA- Quick sequential organ failure assessment score, NEWS2- National early warning score 2, CCI- Charlson Comorbidity Index

‡ Cox regression analyses used except were indicated

a Wilcoxon rank sum test

b Chi-square test

§ Follow up time in 100 person-hours. δ Rate per 100 person-hours.
* Associations with some evidence against the null.

Scenario	Case Details	Problem Encountered	Potential Complications	POCT Result	Impact of POCT
.Lung Transplant patient presents with small bowel obstruction	No symptoms of clinical or biochemical features of CoVID. Required ITU support. CT imaging of Chest reported as Indeterminate for CoVID (not highly suggestive however could not exclude disease).	Given immunosuppression and CT findings, opinion of local Infectious Diseases Team was that difficult to exclude CoVID without PCR testing.	Immunosuppressed patient on Tacrolimus being isolated in area of ITU designated for CoVID. In side room however not positive pressure and potential aerosol generating procedures being down in adjacent bays, therefore at increased risk of exposure. TAT for laboratory PCR 5 days at this time	Negative	Result available within two hours. Prevented unnecessary exposure of patient concerned and ensured safety of other patients nearby in non CoVID area.
Patient with Chest pain	Chest pain radiating down left, raised Troponin, Normal ECG. Clinical team concerned about possible dissection, required CT Aortogram.	Wife was symptomatic with sore throat for past few days and the patient had been sneezing but no fever, tested locally but no result available.	Patient required CT Aortogram which potentially may have been delayed whilst awaiting for result	Negative	Allowed for CT aortogram to be protocolled without enhanced infection control concerns
Patient with Complete heart block and possible CoVID symptoms	Noted to have Complete heart block requiring admission. Was also symptomatic with respiratory symptoms- CoVID could not be excluded.	Required Monitored space and CoVID PCR test. Limited side rooms in trust with monitored space.	Potential long stay in A+E whilst bed became available. This would lead to delay in care for other patients	Negative	A negative POCT Test allowed for the patient to be cohorted in specialised area with minimal impact on care. It also reduced any delay in the patient receiving a pacemaker.

Supplementary Table 5, related to table 3: Selected vignettes indicating utility of POC testing, particularly highlighting importance of negative SARS-CoV-2 tests

Data S1: Clinical Study Protocol for COVIDx study, related to STAR Methods

Study Title:	COVIDx Study: Evaluation of novel diagnostic tests for 2019-nCOV
Protocol Version:	3.0

Chief Investigator:	Prof. Ravi Gupta
CI Address:	Department of Medicine,<br>University of Cambridge,<br>Box 279 (S4),<br>Cambridge Biomedical Campus,<br>Cambridge CB2 0QQ
CI Telephone:	+44 1223 331497<br>+44 1223 331491
Sponsor:	Cambridge University Hospitals NHS Foundation Trust and<br>The University of Cambridge,<br>R&D Department,<br>Box 277,<br>Addenbrooke's Hospital,<br>Hills Road,<br>Cambridge, CB2 0QQ
Safety Reporting:	COVIDx Study Coordinator<br>Cambridge Clinical Trials Unit - Cancer Theme<br>Box 279 (S4), Addenbrooke's Hospital<br>Cambridge Biomedical Campus<br>Hills Road

Cambridge, CB2 0QQ
Email: CCTU.cancer@addenbrookes.nhs.uk
Telephone: 01223 349707

1 Protocol Signatures:

I give my approval for the attached protocol entitled COVIDx Study: Evaluation of novel diagnostic tests for 2019-nCOV dated 21st April 2020.

Chief Investigator

Name: Professor Ravi Gupta

Signature: 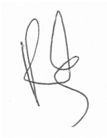

Date: 21.4.20

I agree to comply with the conditions and principles of Good Clinical Practice as outlined in the European Clinical Trials Directives 2001/20/EC and 2005/28/EC, the Medicines for Human Use (Clinical Trials) Regulations 2004 (SI 2004/1031) and any subsequent amendments of the clinical trial regulations, the Sponsor's SOPs, and other regulatory requirements as amended.

I agree to ensure that the confidential information contained in this document will not be used for any other purpose other than the evaluation or conduct of the clinical investigation without the prior written consent of the Sponsor

Principal Investigator

Name: Professor Ravi Gupta

Signature: 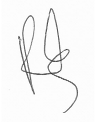

Date: 21.4.2020

2 Study Management Committee(s) and Protocol Contributors

**Sponsor representative**	Prof John Bradley	Cambridge University Hospitals NHS Foundation Trust (CUH)<br>research@addenbrookes.nhs.uk
**Chief Investigator**	Prof Ravi Gupta	Cambridge Institute for Therapeutic Immunology and Infectious Diseases, University of Cambridge; Cambridge University Hospitals NHS Foundation Trust; Rkg20@cam.ac.uk
**Co investigators**	Dr Helen Lee<br><br><br>Prof Paul Lehner<br><br><br>Prof Gordon Dougan<br><br><br>Dr James Whitehorn<br><br><br>Dr Hoi Ping Mok<br><br><br>Dr Dami Collier<br><br><br>Dr. Mike Weekes<br><br>Dr. Hongyi Zhang<br><br>Dr. Nicholas Brown	Diagnostics for the Real World;<br>Chesterford Business Park<br>hl207@cam.ac.uk<br><br>Cambridge Institute for Therapeutic Immunology and Infectious Diseases, University of Cambridge; Cambridge University Hospitals NHS Foundation Trust; pjl30@cam.ac.uk<br><br>Cambridge Institute for Therapeutic Immunology and Infectious Diseases, University of Cambridge; Cambridge University Hospitals NHS Foundation Trust; gd312@cam.ac.uk<br><br>Cambridge University Hospitals NHS Foundation<br><br><br>Cambridge University Hospitals NHS Trust;<br>hoiping.mok@addenbrookes.nhs.uk<br><br><br>Cambridge Institute for Therapeutic Immunology and Infectious Diseases, University of Cambridge<br>adc82@cam.ac.uk<br><br>Cambridge Institute for Therapeutic Immunology and Infectious Diseases, University of Cambridge<br><br>Department of Virology, Cambridge University Hospitals NHS Trust<br>medzhang@doctors.org.uk

		Department of Microbiology, Cambridge University Hospitals NHS Trust<br>nicholas.brown@addenbrookes.nhs.uk
**Clinical Trial Management**	Richard Skells<br>Anne-Laure Vallier<br>Richard Hardy	Cambridge Clinical Trials Unit – Cancer Theme<br>CCTU.cancer@addenbrookes.nhs.uk
**Lead Statistician**	Nikos Demiris	Cambridge Clinical Trials Unit – Cancer Theme<br>CCTU.cancer@addenbrookes.nhs.uk

Table of Contents

3 Abbreviations

AE/AR	Adverse event/Adverse Reaction
CRF	Case Report Form
CTIMP	Clinical Trial of Investigational Medicinal Product
CUH	Cambridge University Hospitals NHS Foundation Trust
DRW	Diagnostics for the Real World Ltd.
GP	General Practitioner
GCP	Good Clinical Practice
HCW	Healthcare Workers
ICF	Informed Consent Form
NIHR	National Institute for Healthcare Research
NPV	Negative Predictive Value
PHE	Public Health England
PIS	Participant Information Sheet
POC	Point of Care
PPE	Personal Protective Equipment
PPV	Positive Predictive Value

R&D	Research and Development
RT-PCR	Reverse Transcription - Polymerase Chain Reaction
REC	Research Ethics Committee
SAE/SAR	Serious Adverse Event/Serious Adverse Reaction
SMG	Study Management Group

4 Study Synopsis

Title of study	COVIDx Study: Evaluation of novel diagnostic tests for 2019-nCOV
Sponsor name	**Cambridge University Hospitals NHS Foundation Trust and the University of Cambridge**
Patient population	Cohort 1: Patients meeting clinically suspected COVID-19 case criteria Cohort 2: CUH Staff in high-risk COVID-19 areas in hospital
Study Design	Cohort 1: COVID-19 hospital patients<br>Cross-sectional study for test 1<br>Case-control study for test 2<br><br>Cohort 2: Healthcare workers<br>Cross-sectional study
Purpose of clinical study	Evaluation of novel diagnostic tests for 2019-nCOV.
Primary objective	Cohort 1: COVID-19 hospital patients<br>**Measure the diagnostic accuracy of two point of care diagnostic tests alone or in combination for COVID in hospital inpatients.**<br>**Part 1. Prospective SAMBA SARS-CoV-2 Point of Care (POC) molecular test**<br>**Part 2. Prospective testing of stored and/or finger prick samples with Prometheus or other 2019-nCOV IgG/IgM test Cassette**<br><br>Cohort 2: Healthcare workers<br>**Determine the prevalence of COVID-19 positivity amongst asymptomatic, pauci-symptomatic and symptomatic healthcare workers over time using SAMBA SARS-CoV-2 POC test and Prometheus or other 2019-nCOV IgG/IgM test Cassette**

	.
Secondary objective (s)	Cohort 1: COVID-19 hospital patients<br>**1. Immune correlates of severe disease**<br>**2. Acceptability to participants of SAMBA.**<br><br>Cohort 2: Healthcare workers<br>**1. Acceptability to participants of each test.**<br>**2. Determine the transmission dynamics of SARS-CoV-2 by phylogenetic analyses.**<br>**3. Assess the impact of these rapid diagnostics on staff absence.**<br>**4. The mental health and welfare of healthcare workers**
Study Outcome Measures	**Cohort 1- COVID-19 hospital patients**<br>1. Sensitivity<br>2. Specificity<br>3. Positive predictive value (PPV)<br>4. Negative predictive value (NPV)<br>Both tests will be measured alone and together against a reference standard (described in main protocol) with interval between start of symptoms and testing noted.<br><br>**Cohort 2- Healthcare workers**<br>1. Prevalence of active COVID-19 infection as determined by a positive SAMBA POC or positive Prometheus 2019-nCOV IgM test, or both.<br>2. Prevalence of past COVID-19 infection as determined by a positive IgG test

	3. Acceptability of testing, objective measures of mental health and staffing levels
Sample Size	**Cohort 1- COVID-19 hospital patients**<br><br>PART 1: SAMBA SARS-CoV-2 molecular POC test<br>We assume a target sensitivity of 0.95 and disease prevalence in the population of 10%. Using a 5% significance level and allowing for an error of 10% gives a required sample size of 182. We will aim to recruit 200 participants to allow for an approximate 10% loss to follow up.<br><br>PART 2: Prometheus 2019-nCOV IgG/IgM test Cassette: We assume a target sensitivity of 0.95. Using a 5% significance level and allowing for an error of 10% gives a required sample size of 186 (93 cases and 93 controls).<br><br>**Cohort 2- Healthcare workers**<br>We assume a target sensitivity of 0.95 and COVID-19 disease prevalence of 30% since this group is expected to have a higher rate than the general population. Using a 5% significance level and allowing for an error of 7% gives a required sample size of 125. We will aim to recruit 150 participants to allow for an approximate 15% loss to follow up. If the actual prevalence is 25% or 35% then 149 or 107 results will be needed respectively.

Summary of eligibility criteria	**Cohort 1- COVID-19 hospital patients**<br>**Inclusion Criteria:**<br>Have given written informed consent to participate<br>Be aged 16 years or older<br>Requiring hospital admission<br>AND<br>Symptomatic of COVID-19 (by clinical or radiological demonstration) in investigator's opinion, which may include any of the following;<br>• Clinical or radiological evidence pneumonia<br>• acute respiratory distress syndrome<br>• influenza like illness<br>• fever ≥37.8°C<br>• acute onset persistent cough (with or without sputum), hoarseness, nasal discharge or congestion, shortness of breath, sore throat, wheezing or sneezing<br>• any other symptom known to be indicative of COVID-19 episode<br><br>Exclusion Criteria:<br>Those below the age of 16 years and those who have not had the standard PHE test applied NB: The SAMBA swab must be taken within 18 hours of the standard laboratory swab<br>Unwilling or unable to comply with study swabbing procedures<br><br>**Cohort 2- Healthcare workers**<br>Inclusion Criteria:

	Have given written informed consent to participate<br>Be aged 16 years or older<br>Healthcare workers on high risk wards at Cambridge University Hospital.<br><br>Exclusion Criteria:<br>Unwilling or unable to comply with study swabbing procedures
Procedures: Screening & enrolment	**Cohort 1- COVID-19 hospital patients**<br><br><u>PART 1: SAMBA SARS-CoV-2 POC test</u><br>Participants will be identified by liaising with nurse and consultant in charge on the shift on any ward with suspected COVID-19 cases. Participants will be screened to ensure they have had or will have the standard hospital COVID-19 test done. The eligibility criteria of all referred participants will be verified and informed consent obtained before enrolment into the study. Written informed will be obtained were appropriate but verbal consent will be obtained in the case of infection control concerns regarding paper in COVID-19 areas. In the case of any incapacitated individuals who are admitted in extremis and are unable to give informed consent because they are in distress, peri-arrest, intubated and ventilated rapidly, or have a pre-existing mental health issue, it is deemed that a diagnoses and appropriate treatment will be in their best interest. Therefore, consent would be sought from their nominated consultee which will be the doctor in charge of

	their care. This is in line with the Medicines for Human Use (Clinical Trials) Regulations 2004.<br><br><u>PART 2: Prometheus 2019-nCOV IgG/IgM test Cassette</u><br>Possible cases of COVID -19 as identified above will have their diagnosis confirmed with the SAMBA SARS-CoV-2 POC test and the diagnostic molecular laboratory standard test. It is recognised that an antibody reactive test result may not be confirmed by molecular assays and an alternative serology test (soon to be available) will be needed to identify false positive results. Residual saved serum in the diagnostic laboratory at CUH will be identified. Each case will be age and sex matched with COVID-19 negative individuals who also has some saved serum in the laboratory. In some COVID-19 positive participants with serial serum samples collected and saved, time to serological test positivity will be documented.<br><br>**Cohort 2- Healthcare workers**<br><br>All healthcare works- doctors, nurses, healthcare assistants, cleaners, catering staff or allied services dedicated to the ward will be approached to participate.
Procedures: Baseline (or on admission, post-screening)	**Cohort 1- COVID-19 hospital patients**<br><br><u>PART 1: SAMBA SARS-CoV-2 POC</u>

	The specimen to be collected will be a combined throat and nasal swab or a swab of endotracheal aspirate. In cases where a combined swab is unable to be obtained, a single swab will be acceptable (with documented justification). These specimens will be collected with the appropriate collection swab and put directly into SAMBA SARS-CoV-2 Buffer in a closed vial. Study staff undertaking the sample collection will wear the appropriate personal protective equipment (PPE) for the risk exposure at all times. Generally, this will be a fluid resistant surgical face mask, a pair of safety glasses, a pair of gloves and a plastic apron. If sampling from an intubated participant, an FFP3 facemask, eye protection, gloves and an apron will be worn. Specimen will be taken and tested shortly after collection at room temp.<br><br><u>PART 2: Prometheus 2019-nCOV IgG/IgM test Cassette</u><br>**After the swab for SAMBA is taken a research nurse will do the finger prick antibody test. The study team will also liaise with the diagnostic lab to retrieve residual saved serum for patients who have tested positive for SARS-CoV-2 using molecular tests at CUH and controls in the study. Cases will be aged-matched (within 5 years) and sex-matched to controls. Saved serum is stored frozen prior to the antibody test (any serum stored at 4^oC must be used within 2 days after collection). 10ul of serum will be extracted from the saved serum in the diagnostic lab and applied to the test kit.**	

	**This will be read at ambient room temperature. In some COVID-19 positive participants with serial sera collected and saved, this test will be applied daily until it becomes positive or until the participant is discharged. Stored serum may be used in in-vitro studies to investigate immune responses to COVID 19.**<br><br>**Cohort 2- Healthcare workers**<br><br>**Two types of specimen will be collected. A combined throat and nasal swab and a finger prick test capillary blood. The swab specimen will be tested with the SAMBA SARS-CoV-2 POC test whilst the finger-prick capillary sample will be tested with the Prometheus 2019-nCOV IgG/IgM test Cassette.**<br><br>**Healthcare workers recruited to this study may be invited to complete a questionnaire (via a link to a website) that aims to assess anxiety levels due to COVID-19.**<br>**This questionnaire will also be sent to other groups of CUH employees including but not limited to:**<br>• **Healthcare workers working on low risk wards**<br>• Healthcare workers who have symptoms themselves<br>• Healthcare workers whose close home contacts have symptoms<br>• **Non-patient facing employees**<br>• **NHS employees working from home**	

Procedures: Inpatient stay & follow up	**Cohort 1- COVID-19 hospital patients**<br><br>Routine laboratory tests and radiology data will be collected from the patient medical records for the CRF.<br><br>Clinical outcome at day 28 will be assessed either by a telephone call to the participant or their GP.<br><br>Acceptability of the test will be assessed at study exit.<br><br>**Cohort 2- Healthcare workers**<br><br>Result of the test will be communicated to the participant and the ward manager. Positive tests will require a further confirmatory PHE COVID-19 prior to following standard protocols.<br><br>Participants that test negative in the initial tests may be retested if clinically indicated.<br><br>Acceptability of each test will be assessed at study exit.<br><br>Staffing levels on a comparable ward in the same hospital that did not receive the intervention will be assessed in order to measure the impact of the POC test on absence from work.

Procedures: End of study	**Cohort 1:** Clinical outcome at day 28 will be assessed either by a telephone call to the participant or their GP. End of participation is defined by the reporting of 28 day status or the last serum blood test, whichever is the later<br><br>**Cohort 2:** Clinical outcome at day 28 will be assessed either by a telephone call to the participant or their GP.<br>**Both a nucleic acid amplification test (SAMBA or other test) and the Prometheus 2019-nCOV IgG/IgM test Cassette will be repeated at day 28 and after 6 months**
Evaluable patients	All participants will be used to assess the study outcomes unless the following occur;<br>• Inadequate samples or radiological data<br>• Withdrawal of consent to allow any prior data collected to be used for the study.

PATIENT ADMISSION
Possible case of COVID

ELIGIBILITY CHECKS

1- Requiring hospital admission

AND

Clinical or radiological evidence of suspected COVID-19, by symptom and medical history assessment plus Investigator judgement

2- Standard samples collected and sent to virology

3- CXR or CT

Hospital standard PPE and isolation procedures apply

REGISTRATION (Day 1)

Collection **SWABS** for **SAMBA tests, Prometheus** and **DATA**

24-72 hours later review standard COVID **STANDARD RESULTS**

Positive results

Daily antibody test*

*up to 40 days of hospitalisation

FOLLOW-UP (Day 28)

Negative results

End of trial

HEALTHCARE WORKERS
Covering wards at risk*

*TMG to define wards at risk

ELIGIBILITY CHECKS

- Healthcare worker on a high-risk ward* at Cambridge University Hospitals. This includes any designated healthcare and allied healthcare worker on the ward.

REGISTRATION (Day 1

Collection **SWABS** for **SAMBA test**
FINGER-PRICK for Prometheus IgG/IgM test
DATA

2h **RESULTS**

Negative results or immune participants*
To carry on with work

Re-test if clinically indicated

Positive results

Confirm with standard reference test
To self-isolate

FOLLOW-UP (Day 28 & 6 Months
Repeat of nucleic acid amplification test (SAMBA or other test) and Prometheus IgG/IgM test

6 Introduction

6.1 Background

The 2019-nCOV originated in the Wuhan China and has since spread to 159 countries around the world (University, 2020). It was declared a pandemic by the World Health Organisation on the 11th of March 2020(Organisation, 2020). The cases in the United Kingdom continue to increase exponentially with up to 5 683 people diagnosed as on the 22nd of March 2020(England, 2020). It is estimated that 1 in 5 people diagnosed will require hospital admission and 1 in 20 intensive care treatment (Guan et al., 2020). The case fatality rate in China was 1.4%(Guan et al., 2020) but higher in some settings such as Italy(Livingston and Bucher, 2020). The case mortality rate in the UK is currently 4.9%(England, 2020). The true mortality rate is however unknown given that we do not know the prevalence of asymptomatic or pauci-asymptomatic infection in the population.

It has been of paramount importance to develop and evaluate diagnostic tests during this pandemic for many reasons. Firstly, to diagnose infected cases, so they may be treated appropriately. Secondly, to identify cases in order to quarantine and stop transmission. Thirdly, to characterise the immune status of those who have and who have not been infected. Both the point-of-care (POC) molecular diagnostics tests and serology tests have the potential to address these questions.

The standard diagnostic test for 2019-nCOV in the UK is done by real-time RT-PCR of the RdRp gene (Corman et al., 2020). Although found to be highly sensitive and specific in assay development when evaluated on in-vitro transcribed RNA of the 2019-nCOV, its diagnostic accuracy in the real-world setting is unknown. In addition, this test is done in only six regional laboratories in England and as 2019-nCOV is a hazard group 3, airborne pathogen, it requires containment level 3 facilities to process the test samples. This causes obvious bottlenecks and in addition to the sheer number of samples that require processing, the regional laboratories are at full capacity and the current turnaround time for test is 24-48 hours. This means patients requiring admission with possible COVID-19 maybe unnecessary isolated or inappropriately cohorted in a COVID ward. A rapid POC test is very much needed.

In addition, in the midst of this public health emergency, every frontline healthcare worker is needed to treat and support acutely unwell patients in NHS hospitals. A major concern is the potential loss of healthcare workers, either through illness or the requirement to self-isolate should a member of their household become unwell. It is critically important to determine early on if healthcare workers are infected with SARS-CoV-2 or not in order to either appropriately self-isolate to prevent transmission in hospitals or remain at work if uninfected or return to work if they are already immune having previously contracted COVID-19. The results of these studies will inform work force planning in this critical time.

6.2 Clinical Data

<u>SAMBA SARS-CoV-2 Point of Care test</u>

The SAMBA II Assay Module and Tablet Module devices were self-declared by the manufacturer (Diagnostics for the Real World - DRW) in conformity of IVD requirements on 10th March 2020, for use as a Nucleic Acid processor + accessories + consumables + software.

The SAMBA II SARS-CoV-2 Test has a limit of detection of 250 cp/ml using serial dilutions of SARS-CoV-2 RNA (2019-nCoV/Italy-INMI1 from EVAg, 1.0E+06 copies/mL) and has been shown to be specific when tested with hCoV-NI63, hCoV-229E, hCoV-OC43, MERC-CoV and SARS-CoV (Coronavirus RNA Specificity Panel form EVAg) as well as combined nasopharyngeal/throat swabs from 30 apparently healthy individuals.

The test was evaluated further on 50 confirmed positive cases and 50 confirmed negative cases of COVID-19 from the UK epidemic. Through statistical analyses, the sensitivity of the assessed test is 98% and the specificity is 100%.

<u>Prometheus 2019-nCOV IgG/IgM test Cassette</u>
The 2019-nCOV IgG/IgM Test Cassette (Whole Blood/Serum/Plasma), Model 25 test/box was granted IVD registration on the 10th March 2020 as an IVD (ref. IVD001099) to place onto the EEA market for use in immunochromatography and principle of Capture ELISA to qualitatively detect 2019-nCOV IgG/IgM antibodies in human serum (or plasma, or whole blood), manufactured by Prometheus Bio Inc.

This kit was evaluated at Zheijang designated admission hospital. In this clinical study, a total of 225 samples were tested, including 105 confirmed samples of novel coronavirus and 120 negative samples.

The results showed that among the 105 positive samples, 1 case was inconsistent according to the comparison of test kit results, while the results of 120 negative cases were all in conformance according to the comparison of test kit results. Through statistical analyses, the sensitivity of the assessed kits is 99.05%, the specificity is 100%, the false positive rate is 0%, the false negative rate is 0.95%, and the total conformity rate is 99.56%. It is quite surprising that apparently, all cases confirmed positive by molecular assay were also reactive with the serology. This does not take into account the 7-10 day window period of infection prior to the development of IgM then IgG antibodies. The results mentioned might only have been obtained if the serologically tested samples were collected as follow-up of cases identified by genomic amplification.

7 Rationale for Study

Our hypothesis is that the Point of care (POC) testing for COVID-19 is comparable to the existing Public Health England (PHE) RT-PCR based test and provides an accurate rapid diagnostic test for COVID-19 which will be tested on patients presenting with COVID19 symptoms at the time of their admission
Our secondary hypothesis is that the Prometheus 2019-nCOV IgG/IgM test Cassette is also a reliable test which in this public health emergency, can serve a role in informing work force planning in this critical time.

8 Study Design

8.1 Statement of Design

This is a prospective, single-centre, diagnostic accuracy study.

<u>Cohort 1- COVID-19 hospital patients</u>
Cross-sectional study for SAMBA II Isothermal PCR-testing

Case-control study for Prometheus 2019-nCOV IgG/IgM test Cassette

Cohort 2- Healthcare workers
Cross-sectional study

8.1.1 Explanation on cohorts

The proposed study will be conducted across two cohorts.

Cohort 1 will be to test the diagnostic accuracy of the point-of-care (POC) tests in possible COVID-19 cases admitted to hospital, or as an existing inpatient.

The POC tests must be validated and proven to save time whilst maintaining accuracy and specificity prior to recruitment to Cohort 2, where decisions to self-isolate or treat may be made.

Cohort 2 is planned in the Healthcare worker (HCW) population and will be embarked upon following review of the outcomes of the trialled tests in Cohort 1, by a broad study management team which will review the clinical study data in liaison with the statistician.

Cohort 2 may be opened to recruitment after or during the recruitment of cohort 1.

8.2 Number of Centres

This is a single-centre study, conducted at Addenbrooke's Hospital in Cambridge.

8.3 Number of Participants

182 evaluable participants are required to complete Cohort 1- Part 1 of this study. We anticipate this will require and extra 18 (200) participants to be registered; however we will continue to recruit participants until target participant completion is achieved.

186 (93 cases and 93 controls). are required to complete Cohort 1- Part 2 of this study. We will continue to recruit participants until target participant completion is achieved.

125 evaluable participants are required to complete Cohort 2 of this study. We anticipate this will require and extra 25 (150) participants to be registered; however we will continue to recruit participants until target participant completion is achieved.

The definition of an evaluable patient is contained in section 9.4

8.4 Participants Study Duration

8.4.1 Cohort 1- COVID-19 hospital patients

The study duration will be a one-off face-to-face visit for most participants with some remote data collection for follow-up. Some patients will have daily sequential testing of residual saved sera for a maximum of 40 days. Follow-up will be made at day 28 via direct contact (telephone) or via their GP or medical records (EPIC) to record their clinical outcome.

Participants will be in study from the date of informed consent until whichever is the later of 28 days after enrolment or the day they are discharged from hospital.

8.4.2 Cohort 2- Healthcare workers

The study duration will be up to three face-to-face visits for most participants. One visit to enrol and perform the tests and two further visits to repeat the tests at day 28 and after six months. In addition, participants that test negative at initial testing may have tests repeated between day 1 and day 28, if clinically indicated. Participants will be informed of results of the tests by email if negative and by phone call if positive. Follow-up for clinical outcome will be made at day 28 via direct contact (face to face, telephone) or via their GP.

8.5 Study Objectives

8.5.1 Clinical Primary objective

8.5.1.1 Cohort 1- suspected COVID-19 hospital patients

Measure the diagnostic accuracy of two point of care diagnostic tests for COVID in hospital inpatients.

1. **SAMBA COVID-19 Point of Care test**
2. **Prometheus 2019-nCOV IgG/IgM test Cassette on stored serum and/or finger prick**
3. **Combined accuracy of both POC tests together**

8.5.1.2 Cohort 2- Healthcare workers

Determine the prevalence of COVID-19 positivity amongst asymptomatic, pauci-symptomatic and symptomatic healthcare workers over time using point of care (POC) rapid diagnostic tests.

8.5.2 Secondary objective

8.5.2.1 Cohort 1- Suspected COVID-19 hospital patients

1. To compare the time from sample acquisition to receipt of result for SAMBA point of care testing and clinical testing through PHE laboratories at Addenbrooke's Hospital
2. **Acceptability to participants of both tests.**
3. **Time to IgM/IgG test positivity for test 2**
4. **Immune correlates of severe disease**

8.5.2.2 Cohort 2- Healthcare workers

1. **Determine the transmission dynamics of COVID-19 by phylogenetic analyses.**
2. **Acceptability to participants of both tests.**
3. **Assess the impact of these rapid diagnostics on staff absence.**
4. **Assess the mental health and welfare of healthcare workers**

8.6 Study Outcome Measures

8.6.1 Primary outcome measure

8.6.1.1 Cohort 1 – Suspected COVID-19 hospital patients

The sensitivity of POC diagnostic tests.

Discrepant analysis of SAMBA POC will be carried out using a mutually agreed alternate gold standard molecular tests. Radiological test and an alternative RT-PCR based test developed and validated by PHE staff at CUH will be included in the analyses. Since positivity of molecular test precedes by several days the development of IgM followed by

IgG antibodies (window period of 7-10 days), the time elapsed between identification of symptoms and test positivity will be carefully monitored for each test. If samples are collected shortly after occurrence of symptoms, it is likely that many patients will be found negative with serology. In contrast, if these samples are collected more than a week after development of symptoms, the POC molecular assay might be negative. Defining the timing of sampling is therefore critical and will be carefully defined. This is also the rationale for assessing the accuracy of both tests in combination.

8.6.1.2 Cohort 2 - Healthcare workers

1. Prevalence of active COVID-19 infection as determined by a positive SAMBA POC or positive Prometheus 2019-nCOV IgM test

2. Prevalence of past COVID-19 infection as determined by a positive Prometheus 2019-nCOV IgG test

8.6.2 Secondary outcome measure

8.6.2.1 Cohort 1 – Suspected COVID-19 hospital patients

1. Other measurement of diagnostic accuracy: specificity, positive predictive value (PPV), negative predictive value (NPV). These are tested against a composite reference standard

2. Time to test result availability for clinical decision making.

3. Time from initial occurrence of symptoms and positive test result for SAMBA-CoV2 and Prometheus IgM or IgG positivity.

4. Clinical outcome at 28 days.

5. Acceptability of point of care test.

8.6.2.2 Cohort 2 - Healthcare workers

1. **Description of the transmission dynamics of COVID-19 in the hospital setting.**

2. Clinical outcome at 28 days and six months and prevalence over time.

3. **The impact of POC testing for COVID-19 on HCW absence compared with a similar ward with no POC COVID testing.**

4. **Acceptability to participants of both tests.**

5. **Objective measures of mental health and staffing levels**

9 Selection and withdrawal of participants

9.1 Inclusion Criteria

9.1.1 Cohort 1 – Suspected COVID-19 hospital patients

To be included in the study the participant must:

- Have given written informed consent to participate
- Be aged 16 years or older
- Criteria for a possible inpatient COVID-19 case;

  Requiring hospital admission
  AND
  Symptomatic of COVID-19 (by clinical or radiological demonstration) in investigator's opinion, which may include any of the following;

  - Clinical or radiological evidence pneumonia
  - acute respiratory distress syndrome
  - influenza like illness
  - fever ≥37.8°C
  - acute onset persistent cough (with or without sputum), hoarseness, nasal discharge or congestion, shortness of breath, sore throat, wheezing or sneezing
  - any other symptom known to be indicative of COVID-19 episode

9.1.2 Cohort 2 - Healthcare workers

To be included in the study the participant must:

- Have given written informed consent to participate
- Be aged 16 years or older.
- Be a healthcare worker on a high-risk ward* at Cambridge University Hospitals. This includes any designated healthcare and allied healthcare worker on the ward.
  *A list of high-risk wards will be provided by the TMG at the start of the recruitment of the cohort 2 and will be kept undated until the end of the recruitment of this same cohort.

9.2 Exclusion Criteria

9.2.1 Cohort 1 - Suspected COVID-19 hospital patients

The presence of any of the following will preclude participant inclusion:

- Those who have not had the standard PHE RT-PCR based COVID-19 test. **NB: The SAMBA swab must be taken within 18 hours of the standard laboratory swab.**
- Unwilling or unable to comply with study swabbing procedures

9.2.2 Cohort 2 - Healthcare workers

The presence of any of the following will preclude participant inclusion:

- Unwilling or unable to comply with study swabbing procedures

9.3 Participant Withdrawal and Replacement Criteria

Patients may withdraw from the study at any time at their own request, without prejudice to further treatment; or may be withdrawn from the study at any time at the discretion of the Investigator or Sponsor for safety, behavioural or administrative reasons.

With the participants' consent, any samples collected as part of this study prior to participant withdrawal will be retained, analysed and used by the study team. However, if a participant requests that their sample(s) are destroyed, this will be undertaken by the study team.

Both non-evaluable participants and participants who withdraw from the study prior to blood and tissue sample collection will be replaced. Evaluability criteria is outlined in the section below.

9.4 Evaluability Criteria

All participants will be used to assess the study outcomes unless the following occur;

- Inadequate samples or radiological data
- Withdrawal of consent to allow any prior data collected to be used for the study.

10 Procedures and Assessments

10.1 Participant Identification (on day of admission)

10.1.1 Cohort 1- Suspected COVID-19 hospital patients

10.1.1.1 SAMBA SARS-CoV-2 Point of Care test

Participants will be identified by liaising with medical staff in charge on the shift in any department receiving suspect cases of COVID-19. Participants will be approached by a designated member of the study team and screened to ensure they have had the standard PHE RT-PCR based COVID-19 test done.

The eligibility criteria of all referred participants will be verified and informed consent obtained before enrolment into the study.

Informed consent will be obtained from patients. However, incapacitated individual unable to give informed consent because they are in distress, peri-arrest, intubated and ventilated rapidly or have a pre-existing mental health issue, will be represented by a medical staff from their care team. If this person decides that it is in the patient best interest to take part into this study, they will be allowed to consent on behalf of the patient as their nominated consultee. This is in line with the Medicines for Human Use (Clinical Trials) Regulations 2004.

10.1.1.2 Prometheus 2019-nCOV IgG/IgM test Cassette

Patients deemed COVID-19 positive with the SAMBA COVID POC test and the diagnostic laboratory PHE RT-PCR based COVID-19 standard test will have their residual serum collected from the diagnostic laboratory at CUH.
For these COVID-19 positive participants with serial serum samples collected and saved, time to serological test positivity will be documented but using any excess blood samples taken during their inpatient stay up to 40 days post consent. Stored serum may also be used in in-vitro studies to investigate immune responses to COVID 19.

10.1.2 Cohort 2- Healthcare workers

Most healthcare workers: doctors, nurses, healthcare assistants, cleaners, catering staff or allied services designated to the ward will be approached to participate in this study. Participants will be identified through a senior ward nurse and will be approached by a designated member of the

study team. Staff members who have previously been tested for COVID-19 outside the study can be included.

Cohort 2 will not be embarked upon until the POC tests have been evaluated in Cohort 1 and the study management team reviews the results and agrees the study can progress to this cohort, at which point the tests may be used to guide clinical decision making.

10.2 Consent (on day/within 18 hours of admission or identification within hospital as inpatient)

The Informed Consent form must be approved by the Research Ethics Committee (REC) and must be in compliance with Good Clinical Practice (GCP), local regulatory requirements and legal requirements. The investigator or designee must ensure that each study participant, or his/her legally acceptable representative (nominated consultee), is fully informed about the nature and objectives of the study and possible risks associated with their participation.

The investigator or designee will obtain informed consent (written or verbal) from each participant or the participant's nominated consultee before any study-specific activity is performed. The informed consent form used for this study and any change made during the course of this study, must be prospectively approved by the REC. Where written informed consent is obtained, the investigator will retain the original of each participant signed informed consent form.

In cases whereby the participant is in isolation but NOT incapacitated, and conditions limit the ability for paperwork, verbal participant consent may be taken from the participant. This must be fully documented in the case notes. If possible, a signed consent form should be obtained from the participant at a later date, once isolation has been lifted.

Should a participant require a verbal translation of the study documentation by a locally approved interpreter/translator, it is the responsibility of the individual investigator to use locally approved translators.

The informed consent form used for this study, and any changes made during the course of this study, will be approved by the REC.

Any new information which becomes available, which might affect the participant's willingness to continue participating in the study will be communicated to the participant or their nominated consultee as soon as possible. This will be verbally if they are an inpatient, and via the telephone if they have been discharged. A follow up letter may be posted to the participant depending on the nature of the information to be communicated.

10.3 Screening evaluation (on day/within 18 hours of admission or identification within hospital as inpatient)

10.3.1 Cohort 1

The following screening assessments should be completed before registering a participant to Cohort 1 of the COVIDx study;

- Details of consent (incl. gender, date of consent, age at consent & whether the patient or legal representative gave the consent)
- Date of admission or identification within hospital as an inpatient

- Date and time of occurrence of first symptom(s) leading to hospital admission or identification within hospital as an inpatient
- Standard diagnostic lab PCR-based test (s) for COVID-19 requested
- Details of symptoms (as per eligibility criteria), including temperature (if available, in ^{o}C)

10.3.2 Cohort 2

The following screening assessments should be completed before registering a participant to Cohort 2 of the COVIDx study;

- Details of consent (incl. gender, date of consent & age at consent)
- Work setting details (ward)
- Invitation to complete a questionnaire to assess anxiety levels due to COVID-19

Healthcare workers recruited to this study are those working on high risk wards. In order to compare anxiety levels in this group, to those working in lower risk areas and healthcare workers who have symptoms or close contacts with symptoms an invitation to complete a questionnaire would include other groups of CUH employees as follows:

- **Healthcare workers working on low risk wards**
- Healthcare workers who have symptoms themselves
- Healthcare works whose close home contacts have symptoms
- **Non-patient facing employees**
- **NHS employees working from home**

In addition to questions related to anxiety levels, the questionnaire collects: details about job role (to distinguish between higher and lower risk groups); COVID-19 symptoms in the participant and whether the participant has been in contact with a known case of COVID-19; self isolation; and any previous COVID-19 testing.

Invitations to complete the questionnaire would be sent via a link to a website. Completion of the questionnaire is optional and responses to the questionnaire will be anonymous. Personally identifiable data will not be collected.

10.4 . Participant Registration

Participants must be formally registered into the study once they have given informed consent and meet all the eligibility criteria. To register a participant, the registration page of the electronic Case Report Form (eCRF) must be completed, electronically signed-off by the Principal Investigator or delegated staff member.

A unique Study I.D. will be generated on the system, which will be the means of identifying particular participants between the Cambridge Clinical Trial Unit, Sponsor and site delivery team, in place of any identifiable.

10.5 Post-screening

10.5.1 Cohort 1

The following assessments and procedures should be completed after any Cohort 1 participant has been admitted or identified from the ward, but after they have passed screening;

- Details of admission

- Radiological data collection (type of scan, date of scan and test result)
- SAMBA II Point of Care Test swab to be performed on either a combined nasal and throat approach or endotracheal tube aspirate, or nasopharyngeal swab (as clinically appropriate and accessible). In cases whereby a dual swab is inaccessible, a single swab of the nose or throat is acceptable (with documented justification) **NB: The SAMBA swab must be taken within 18 hours of the standard laboratory swab.**
- Prometheus 2019-nCOV IgG/IgM finger-prick test
- Signs and symptoms (to include a minimum of respiratory rate (breaths per minute), oxygen saturation, PaO_2:FiO_2 ratio, temperature (^oC), heart rate (bpm), blood pressure (systolic and diastolic), sternal capillary refill time of >2 seconds)
- Clinical Laboratory Investigations (to include as a minimum; White blood cell count, lymphocytes count, platelet count, haemoglobin, C-reactive protein, Procalcitonin, Ferritin, Lactate dehydrogenase, Alanine aminotransferase, Aspartate aminotransferase, Creatine kinase, Creatinine, D-dimer, Interleukin assessments (IL-1, IL-6 & IL-8) & TNFα. Results from any repeated laboratory investigations also should be recorded for the duration of the admission).

10.5.2 Cohort 2

The following assessments and procedures should be completed after any Cohort 2 participant has been enrolled, but after they have passed screening;

- SAMBA Point of Care Test swab to be performed on either a combined nasal and throat approach or endotracheal tube aspirate, or nasopharyngeal swab (as clinically appropriate and accessible)
- Prometheus 2019-nCOV IgG/IgM finger-prick test

If SAMBA II testing identifies a positive result for COVID-19 detection, they will be notified alongside their ward manager. A second swab would be taken for confirmatory test and storage (at CL3 containment in the Department of Medicine or Virology) for sequencing of virus; local procedures will apply in terms of isolation.

For participants that test negative, testing can be repeated between day 1 and day 28 if clinically indicated.

10.6 During Inpatient Stay

FOR COHORT 1 ONLY, if SAMBA test is deemed positive

The following assessments should be completed throughout the duration of any Cohort 1 participant's inpatient stay;

- Details of admission (only if there are changes)
- Prometheus 2019-nCOV IgG/IgM test on any excess samples taken routinely (daily if possible), for a maximum of 40 days.
- Prometheus 2019-nCOV IgG/IgM test will also be done on a subset of COVID-19 negative participants, which will act as controls.
- Follow-up at day 28 as per section 10.7

10.7 Follow-up and End of Study Participation & Outcome

COHORT 1

Direct patient involvement will end following the 28 day (or discharge if later than 28 days) follow up outcome status collection. Only remote data will be collected after the first face-to-face visit for Cohort 1 participants. This could be conducted by checking hospital records or contacting their general practitioner.

COHORT 2

Both a nucleic acid amplification test (SAMBA or other test) and the Prometheus 2019-nCOV IgG/IgM test Cassette will be repeated at day 28 and after 6 months. The Samba will be used to see whether the person has cleared virus or become newly positive and the Prometheus test is needed to measure prevalence of immunity in this vital group.

Cohort 2 participants will be contacted directly (face to face, telephone) for clinical outcome at day 28.

10.8 Schedule of Assessments for cohort 1

Assessment	Screening and baseline	Inpatient Stay		After Discharge
Day	Admission	Day 1	Day 28[7]	
Approach & Informed consent[1]	X			
Eligibility Criteria	X			
Participant Registration	X			
Details of Admission		X[2]		
SAMBA **SARS-CoV-2** POC Swab[3]		X		
PHE COVID-19 PCR Swab		X		
Prometheus test cassette		X		
Radiology Data Collection[4]		X		
Signs & Symptoms[5]				
Clinical Laboratory Investigations[6]		X		
Clinical Outcome & COVID Status[8]			X[7]	
Serum Collection for SAMBA positive patients[9]		X[10]		

[1]Approach and Informed Consent must occur before any other activity on the schedule
[2]Details of admission may change through the course of the inpatient stay, and the progress of admission should be reported as such. To include type of isolation on admission and following PHE standard test
[3]Either a combined nose and throat swab, nasopharyngeal swab or an endotracheal tube aspirate sample should be taken, and recorded as such. In cases whereby only a singular nose or throat swab can be taken, due to compliance or inability to access, the reasons should be documented appropriately.
[4]Results to be taken from standard of care X-ray or CT scan, whichever is available. Results from any repeated radiology scans also should be recorded for the duration of the admission
[5]to include a minimum of respiratory rate (breaths per minute), oxygen saturation, $PaO_2:FiO_2$ ratio, temperature (°C), heart rate (bpm), blood pressure (systolic and diastolic), sternal capillary refill time of >2 seconds.
[6]to include as a minimum; White blood cell count, lymphocytes count, platelet count, haemoglobin, C-reactive protein, Procalcitonin, Ferritin, Lactate dehydrogenase, Alanine aminotransferase, Aspartate aminotransferase, Creatine kinase, Creatinine, D-dimer, Interleukin assessments (IL-1, IL-6 & IL-8) & TNFα. Results from any repeated laboratory investigations also should be recorded for the duration of the admission
[7]Clinical outcome and COVID status can be conducted remotely via medical records access if participants are still inpatients at Day 28. If the patient is discharged prior to Day 28, research staff should contact their general practitioner for details.
[8]to include survival status, confirmation of any intensive care requirements and details as such.
[9]From excess routine clinical samples ONLY, as available after standard of care tests are performed.
[10]Serum collection for IgM/IgG analysis ideally should be sought daily from routine samples up to Day 40, and only during inpatient admission.

10.9 Schedule of Assessments for cohort 2

Assessment	Screening and baseline	After registration		Follow-up

Day	Day 1	Day 1	Day 2- Day 27	Day 28[5]	6 months
Approach & Informed consent[1]	X				
Eligibility Criteria	X				
Invitation to complete questionnaire	X				
Participant Registration	X				
Collection of baseline data		X			
SAMBA COVID-19 PCR Swab[2]		X	X[4]		
Prometheus test cassette[3]		X	X[4]		
Clinical Outcome & COVID Status		X		X	
Repeat Testing[5]				X	X
Working Arrangements[6]	X			X	

[1]Approach and Informed Consent must occur before any other activity on the schedule
[2]Either a combined nose and throat swab, nasopharyngeal swab or an endotracheal tube aspirate sample should be taken, and recorded as such
[3]Finger prick capillary whole blood test
[4]Participants that test negative can be retested if clinically indicated
[5]. Both a nucleic acid amplification test (SAMBA or other test) and the Prometheus 2019-nCOV IgG/IgM test Cassette will be repeated at day 28 and after 6 months.
[6]To include details of the ward worked on.

11 Assessment of Safety and Safety Reporting

11.1 Definitions

11.1.1 Adverse event (AE)

Any untoward medical occurrence in a patient or clinical study subject that does not necessarily have a causal relationship with the study procedure. An adverse event can therefore be any unfavourable and unintended sign (including an abnormal laboratory finding), symptom, or disease temporally associated with study procedures.

11.1.2 Serious adverse event or serious adverse reaction (SAE / SAR)

Any untoward medical occurrence that:

1. results in death
2. is life-threatening
3. requires hospitalisation or prolongation of existing hospitalisation
4. results in persistent or significant disability or incapacity
5. is otherwise considered medically significant by the investigator

* Life-threatening in the definition of a serious adverse event or serious adverse reaction refers to an event in which the subject was at risk of death at the time of event; it does not refer to an event which hypothetically might have caused death if it were more severe.

**Hospitalisation is defined as an inpatient admission, regardless of length of stay, even if the hospitalisation is a precautionary measure for continued observation. Hospitalisation for a pre-existing condition, including elective procedures, which has not worsened, does not constitute a serious adverse event.

*** Some medical events may jeopardise the subject or may require an intervention to prevent one of the above characteristics/ consequences. Such events (hereinafter referred to as 'important medical events') should also be considered as 'serious'.

11.2 Expected Adverse Events (AE) for this study

11.2.1 Cohort 1 - COVID-19 hospital patients

In cohort 1, The POC tests will not be used for clinical decision making.

List of expected occurrences related to the study procedures:

- Discomfort from obtaining nasal or throat swab. However, this will be the same as the standard PHE sampling technique and is discomfort that lasts a few seconds.

11.2.2 Cohort 2- Healthcare workers

List of expected occurrences related to the study procedures:

- Discomfort from obtaining nasal or throat swab. However, this will be the same as the standard sampling technique and is discomfort that lasts a few seconds.
- In the event of a false positive being acted on, risks to HCW could include cohorting along with COVID19 patients and potentially receiving antibiotics / antivirals. Or a recommendation for isolation, monitoring of household or other close contacts for symptoms, patient isolation that might limit contact with family or friends.
- In the event of a false negative risks to HCW include delayed appropriate treatment (although no evidence-based treatments are available at present). Risk on on-going transmission.

11.3 Evaluation of adverse events

As the COVIDx study does not involve an Investigational Medicinal Product (IMP), non-serious Adverse Events will not be collected. Only Serious Adverse Events (SAEs) (as defined above) that are suspected to be related to study procedures will be collected.

11.3.1 Assessment of seriousness

Seriousness is assessed against the criteria in section 12.1. This defines whether the event is an adverse event, serious adverse event or a serious adverse reaction.

11.3.2 Assessment of causality

Definitely: A causal relationship is clinically/biologically certain. **This is therefore an Adverse Reaction**.

Probable: A causal relationship is clinically / biologically highly plausible and there is a plausible time sequence between onset of the AE and study procedures. **This is therefore an Adverse Reaction**.

Possible: A causal relationship is clinically / biologically plausible and there is a plausible time sequence between onset of the AE and study procedures. **This is therefore an Adverse Reaction**.

Unlikely: A causal relation is improbable and another documented cause of the AE is most plausible. **This is therefore an Adverse Event**.
Unrelated: A causal relationship can be definitely excluded and another documented cause of the AE is most plausible. **This is therefore an Adverse Event**.

11.3.3 Clinical assessment of severity

Mild: The subject is aware of the event or symptom, but the event or symptom is easily tolerated
Moderate: The subject experiences sufficient discomfort to interfere with or reduce his or her usual level of activity
Severe: Significant impairment of functioning; the subject is unable to carry out usual activities and / or the subject's life is at risk from the event.

All SAEs experienced will be graded for severity according to the NCI CTCAE Toxicity Criteria (Version 4.03). CTCAE v4.03 can be downloaded from the following URL: http://ctep.cancer.gov/reporting/ctc.htmL

11.3.4 Reporting serious adverse events

Only SAEs that are possibly, probably or definitely related will be reported.

The research team needs to complete and sign the SAE form and get it assessed for expectedness and relatedness within 24h by the chief co-chief investigator or his deputy.

If the SAE is deemed to be **related** by the chief investigator or his deputy, the SAE must be notified to the **Sponsor** immediately but not more than 24 hours of first notification.

If the SAE is deemed to be **related** and **unexpected** (i.e. not listed in section 11.2), it must be notified to the Research Ethics Committee within 15 days of first notification using the Health Research Authority report of serious adverse event form (see HRA website).

In the case of an SAE, the subject must be followed-up until clinical recovery is complete and laboratory results have returned to normal, or until the disease has stabilised.

12 Storage and Analysis of Samples

Blood and tissue samples will be collected for research as detailed below. Other analyses may be performed on the samples in line with the study objectives.

12.1.1 Viral transport media

The research swab samples will be stored and analysed for the genomic data on 2019-nCOV. This will be used to explore transmission dynamics in the hospital setting by phylogenetic analyses of 2019-nCOV sequence data.

12.1.2 Saved Serum

Saved serum from the diagnostic lab may be used for in-vitro studies to investigate immune responses to COVID-19.. Unused saved serum will be stored and will be utilised for future unrelated research project with prior ethics approval.

13 Statistics – Evaluation of results

13.1 Statistical methods

Cohort 1- COVID-19 hospital patients

Part 1: SAMBA SARS-CoV-2 Point of Care test: Cross-sectional study

Measurements of diagnostic accuracy of the SAMBA II POC- Sensitivity, specificity, positive predictive value (PPV), negative predictive value (NPV) tested against a dual composite reference standard.

In the absence of a gold standard, a composite alternative is used which includes the result from the current CUH / PHE RT-PCR test for COVID, an alternative RT-PCR based test developed and validated by PHE staff at CUH and chest radiological findings. If at least 2 of these are positive, then this will be the definition of a positive case. This composite alternative significantly reduces the chance of a false positive since 3 positive results from 3 diagnostic tests with individual sensitivity of 0.95 result in a false positive probability of 0.00012, thus effectively comparing to a gold standard.

Part 2: Prometheus 2019-nCOV IgG/IgM test Cassette

Measurements of diagnostic accuracy of the Finger Prick COVID-19 antibody test- Sensitivity, specificity, positive predictive value (PPV), negative predictive value (NPV) tested against a dual composite reference standard.

In this case the composite gold standard will be the result from the current standard RT-PCR test for COVID, an alternative RT-PCR based test developed and validated by PHE staff at CUH chest radiological findings and SAMBA POC test described above. If at least 2 of these are positive, then this will be the definition of a positive case.

Cohort 2- Healthcare workers- Cross-sectional survey

Summary statistics will be used to describe the population. The prevalence of acute and past COVID-19 infection will be expressed as a percentage.

13.2 Number of Participants to be enrolled

Cohort 1- COVID-19 hospital patients

Part 1: SAMBA COVID-19 Point of Care test: Cross-sectional study

We assume a target sensitivity of 0.95 and disease prevalence in the population of 10%. Using a 5% significance level and allowing for an error of 10% gives a required sample size of 182. We will aim to recruit 200 participants to allow for an approximate 10% loss to follow up. Note that this may be a conservative calculation since a potentially higher prevalence will lead to smaller required sample sizes. For example, if the prevalence is 12% or 15% then 152 or 122 results will be needed respectively.

Part 2: Prometheus 2019-nCOV IgG/IgM test Cassette: Case-control study

We assume a target sensitivity of 0.95. Using a 5% significance level and allowing for an error of 10% gives a required sample size of 186 (93 cases and 93 controls).

Cohort 2- Healthcare workers- Cross-sectional survey

We assume a target sensitivity of 0.95 and disease prevalence of 30% since this group is expected to have a higher rate than the general population. Using a 5% significance level and allowing for an error of 7% gives a required sample size of 125. We will aim to recruit 150 participants (50 is each of the strata- asymptomatic, pauci-symptomatic and symptomatic) to allow for an approximate 15% loss to follow up. If the actual prevalence is 25% or 35% then 149 or 107 results will be needed respectively.

13.3 Definition of the end of the study

The definition of the end of study is the last participant's COVID-19 outcome status entered in the database.

14 Data handling and record keeping

14.1 eCRF

All data will be transferred into an electronic Case Report Form (eCRF) which will be anonymised. All study data in the eCRF must be extracted from and be consistent with the relevant source documents. The eCRF must be completed by the investigator or designee in a timely manner. It remains the responsibility of the investigator for the timing, completeness and accuracy of the eCRF. The eCRF will be accessible to study coordinators, data managers, the investigators, clinical study monitors, auditors and inspectors as required. For further information, please refer to the Case Report Form Guidelines document.

14.2 Source Data

To enable monitoring, audit and/or inspection the investigator must agree to keep records of all participating participants (sufficient information to link records e.g. hospital records and samples) and all original signed informed consent form.

Source data include, but are not limited to:

- Participant Medical Records
- Online Medical Records (e.g. medical records, prescribing records, results/reports from clinical investigations such as blood tests or scans)
- Signed and dated informed consent forms
- Worksheets and forms for sample collection, processing storage, shipment and diagnostic test output.

14.3 Data Protection & Participant Confidentiality

All investigators and study site staff involved in this study must comply with the requirements of the Data Protection Act 2018 and Trust Policy with regards to the collection, storage, processing, transfer and disclosure of personal information and will uphold the Act's core principles.

15 Ethical & Regulatory considerations

15.1 Ethical committee review

Before the start of the study or implementation of any amendment we will obtain approval of the study protocol, protocol amendments, informed consent forms and other relevant documents e.g., advertisements and GP information letters if applicable from the REC. All correspondence with the REC will be retained in the Study Master File (TMF) and/or Investigator Site File (ISF).

Annual reports will be submitted to the REC in accordance with national requirements. It is the Chief Investigator's responsibility to produce the annual reports as required.

15.2 Protocol Amendments

Protocol amendments must be reviewed and agreement received from the Sponsor for all proposed amendments prior to submission to the Health Research Authority (HRA) and REC.

The only circumstance in which an amendment may be initiated prior to HRA/REC approval is where the change is necessary to eliminate apparent, immediate risks to the participants (Urgent Safety Measures). In this case, accrual of new participants will be halted until the HRA/REC approval has been obtained.

In the event of an urgent safety measure, the chief investigator (or delegate) will cascade the information verbally and/or by email to each participating site within 24 hours.

15.3 Peer Review

Scientific review of the COVIDx study was arranged by Professor John Bradley (Director of Cambridge BRC & Director of Research at the Sponsor institution).

The study was approved for strategic importance for tackling the COVID-19 pandemic by the COVID Oversight Committee. The protocol approved for scientific value by the Research Advisory Committee. Both committees are embedded into the research infrastructure at Cambridge University Hospitals NHS Foundation Trust.

15.4 Declaration of Helsinki and Good Clinical Practice

The study will be performed in accordance with the spirit and the letter of the declaration of Helsinki, the conditions and principles of Good Clinical Practice, the protocol and applicable local regulatory requirements and laws.

15.5 GCP Training

Although not mandatory for non-CTIMP studies, it is recommended that all study staff should hold evidence of appropriate GCP training or undergo GCP training prior to undertaking any responsibilities on this study. This training should be updated every 2 years or in accordance with your Trust's policy.

16 Sponsorship, Financial and Insurance

The study is sponsored by Cambridge University Hospitals NHS Foundation Trust and the University of Cambridge.

The study will be funded and supported by philanthropic financial and material donations to aid study conduct; Cambridge Biomedical Research Centre (BRC), Diagnostics for the Real World Ltd. (DRW) and staffing funded by other major infrastructure awards.

Cambridge University Hospitals NHS Foundation Trust, as a member of the NHS Clinical Negligence Scheme for Trusts, will accept full financial liability for harm caused to participants in the clinical study caused through the negligence of its employees and honorary contract holders. There are no specific arrangements for compensation should a participant be harmed through participation in the study, but no-one has acted negligently.

The University of Cambridge will arrange insurance for negligent harm caused as a result of protocol design and for non-negligent harm arising through participation in the clinical study.

As there are no additional visits for the COVIDx study, there are no additional provisions for participant's travel or sustenance expenses. All participants will be inpatients during the course of their participation, barring outcome status confirmed remotely.

17 Monitoring, Audit & Inspection

The investigator must make all study documentation and related records available should an inspection occur. Should a monitoring visit or audit be requested, the investigator must make the study documentation and source data available to the Sponsor's representative. All participant data must be handled and treated confidentially.

The Sponsor's monitoring frequency will be determined by an initial risk assessment performed prior to the start of the study. A monitoring plan will be generated detailing the frequency and scope of the monitoring for the study. Throughout the course of the study, the risk assessment will be reviewed and the monitoring frequency adjusted as necessary.

Investigators should try to ensure that data is entered into the database in a timely manner, verified with source data, and that their site files are up to date at all times in order to streamline the monitoring actions of the Sponsor.

18 Protocol Compliance and Breaches of GCP

Prospective, planned deviations or waivers to the protocol are not allowed under the UK regulations on Clinical Studies and must not be used.

Protocol deviations, non-compliances, or breaches are departures from the approved protocol. They can happen at any time, but are not planned. They must be adequately documented on the relevant forms and reported to the Chief Investigator immediately.

Deviations from the protocol which are found to occur constantly again and again will not be accepted and will require immediate action and could potentially be classified as a serious breach.

Any potential/suspected serious breaches of GCP must be reported immediately to the Sponsor without any delay.

19 Publications policy

Ownership of the data arising from this study resides with the study team. On completion of the study the data will be analysed and tabulated and a Final Study Report prepared.

All presentations and publications relating to the study must be authorised by the SMG and any other parties where authorisation forms part of a legally binding funding award or agreement.

It is anticipated any results from this research will be submitted to peer reviewed journals for publication and presented at national and international scientific meetings. Publications will be made Open Access in line with University of Cambridge policies.

Any subsequent publications will acknowledge the support of the National Institute for Healthcare Research (NIHR) Cambridge Biomedical Research Centre.

Participants that wish to be informed of the results of the study will be given a lay summary of results when they are available, post-analysis. Responsibility for requesting the results resides with the participating site study team.

20 References

Corman, V.M., Landt, O., Kaiser, M., Molenkamp, R., Meijer, A., Chu, D.K.W., Bleicker, T., Brunink, S., Schneider, J., Schmidt, M.L.*, et al.* (2020). Detection of 2019 novel coronavirus (2019-nCoV) by real-time RT-PCR. Euro Surveill *25*.

England, P.H. (2020). Total UK COVID-19 Cases Update.

Guan, W.J., Ni, Z.Y., Hu, Y., Liang, W.H., Ou, C.Q., He, J.X., Liu, L., Shan, H., Lei, C.L., Hui, D.S.C.*, et al.* (2020). Clinical Characteristics of Coronavirus Disease 2019 in China. N Engl J Med.

Livingston, E., and Bucher, K. (2020). Coronavirus Disease 2019 (COVID-19) in Italy. JAMA.

Organisation, W.H. (2020). WHO Director-General's opening remarks at the media briefing on COVID-19 - 11 March 2020.

University, J.H. (2020). Coronavirus COVID-19 Global Cases by the Centre for Systems Science and Engineering (CSSE) at John Hopkins University (JHU).
